# Key features of the environment promoting liver cancer in the absence of cirrhosis

**DOI:** 10.1038/s41598-021-96076-2

**Published:** 2021-08-18

**Authors:** Marco Youssef William Zaki, Ahmed Khairallah Mahdi, Gillian Lucinda Patman, Anna Whitehead, João Pais Maurício, Misti Vanette McCain, Despina Televantou, Sameh Abou-Beih, Erik Ramon-Gil, Robyn Watson, Charlotte Cox, Jack Leslie, Caroline Wilson, Olivier Govaere, John Lunec, Derek Austin Mann, Sirintra Nakjang, Fiona Oakley, Ruchi Shukla, Quentin Mark Anstee, Dina Tiniakos, Helen Louise Reeves

**Affiliations:** 1 Faculty of Medical Sciences, Newcastle University Translational and Clinical Research Institute, Newcastle-upon-Tyne, NE2 4HH UK; 2 Faculty of Medical Sciences, Newcastle University Biosciences Institute, Newcastle-upon-Tyne, NE2 4HH UK; 3 Department of Biochemistry, Faculty of Pharmacy, Minia University, Minia, 61519 Egypt; 4 Department of Pathology and Forensic Medicine, College of Medicine, Al-Nahrain University, Baghdad, Iraq; 5 Department of Pathology, Faculty of Medicine, Fayoum University, Fayoum, 63514 Egypt; 6 National and Kapodistrian University of Athens Aretaieion Hospital, Athens, Greece; 7 Liver Unit, Freeman Hospital, Newcastle-Upon-Tyne Hospitals NHS Foundation Trust, Newcastle-upon-Tyne, NE7 7DN UK

**Keywords:** Cancer models, Hepatocellular carcinoma, Non-alcoholic steatohepatitis, Type 2 diabetes, Obesity, Cancer microenvironment

## Abstract

The prevalence of obesity and non-alcoholic fatty liver disease (NAFLD) associated hepatocellular carcinoma (HCC) is rising, even in the absence of cirrhosis. We aimed to develop a murine model that would facilitate further understanding of NAFLD-HCC pathogenesis. A total of 144 C3H/He mice were fed either control or American lifestyle (ALIOS) diet, with or without interventions, for up to 48 weeks of age. Gross, liver histology, immunohistochemistry (IHC) and RNA-sequencing data were interpreted alongside human datasets. The ALIOS diet promoted obesity, elevated liver weight, impaired glucose tolerance, non-alcoholic fatty liver disease (NAFLD) and spontaneous HCC. Liver weight, fasting blood glucose, steatosis, lobular inflammation and lipogranulomas were associated with development of HCC, as were markers of hepatocyte proliferation and DNA damage. An antioxidant diminished cellular injury, fibrosis and DNA damage, but not lobular inflammation, lipogranulomas, proliferation and HCC development. An acquired CD44 phenotype in macrophages was associated with type 2 diabetes and NAFLD-HCC. In this diet induced NASH and HCC (DINAH) model, key features of obesity associated NAFLD-HCC have been reproduced, highlighting roles for hepatic steatosis and proliferation, with the acquisition of lobular inflammation and CD44 positive macrophages in the development of HCC—even in the absence of progressive injury and fibrosis.

## Introduction

Non-alcoholic fatty liver disease (NAFLD) comprises a wide spectrum of pathological features and severity, ranging from mild simple steatosis, to more severe fat deposition combined with necro-inflammatory changes or non-alcoholic steatohepatitis (NASH), to fibrosis and eventually cirrhosis^1^. Cirrhosis is the most important risk factor associated with the development of hepatocellular carcinoma (HCC). NAFLD has an estimated prevalence exceeding 25% of the population globally^2^. It is the liver manifestation of the obesity related metabolic syndrome and is often associated with insulin resistance or type 2 diabetes (T2DM), hypertension and dyslipidaemia^3^. Although the underlying mechanisms are not clearly established, obesity, T2DM and NAFLD—even in the absence of cirrhosis—are all independently associated with an increased risk of developing HCC^4,5^. Attributed to hepatitis B and C infections, HCC is the third most common cause of cancer related mortality worldwide^6^. In some geographical areas, however, NAFLD is the commonest cause, with its incidence expected to continue rising in westernised nations for the next 20–30 years^7,8^. Identifying which patients with the metabolic syndrome are most at risk of developing HCC, as well as understanding how to prevent it, are important needs.

A number of animal models have been used to study the pathogenesis of NAFLD and HCC. These typically involve genetically modified C57Bl/6 mice, although a number also involve dietary modifications. Often, high fat diet alone is insufficient to cause cancers and is given in combination with additional means of promoting oxidative stress/fibrosis or carcinogens^9,10^. These approaches all have value, with the use of C57Bl/6 mice a significant asset lending itself to subsequent genetic manipulation. They may also have limitations. In Wolfe et al., mice developed features of the metabolic syndrome and NAFLD, but tumours were restricted to those receiving a choline deficient diet in association with raised aminotransferases and fibrosis^11^. In Wei et al, over 80% of C57Bl/6 mice fed choline deficient L-amino acid defined (CDAA) diet developed tumours with some features of steatosis and liver injury, but this was not in association with features of the metabolic syndrome^12^. In Asgharpour et al., C57Bl/6 mice crossed with 129S1 (B6/129) fed a western diet did develop obesity, fatty liver injury and severe fibrosis with the majority developing cancers, but in the absence of impaired glucose tolerance^13^. In Dowman et al., 60% of C57Bl/6 fed the American Lifestyle (ALIOS) diet developed small 1–2 mm tumours, associated with features of NASH and fibrosis, although in the absence of obesity or impaired glucose tolerance^14^. In summary, while there are major advantages using C57Bl/6 mice, they do not develop the metabolic syndrome with age, even if they are fed an obesigenic diet. While these models are undoubtedly valuable for studying hepatocarcinogenesis in the presence of the injuries created, they do not necessarily capture the multifactorial processes underlying obesity and NAFLD associated HCC—which in up to 50% of humans, arises in the absence of advanced fibrosis or cirrhosis. Thus, the focus on C57Bl/6 mice may have limited the opportunities to identify all mechanisms relevant to the human metabolic syndrome and NAFLD-HCC. Consequently, we chose the ALIOS diet, but used the C3H/He strain of mouse^15^. C3H/He mice are susceptible to both obesity^16^ and hepatoma development^17^ with age.

## Results

### Exacerbation of the metabolic phenotype of C3H/He mice promotes NAFLD and HCC.

**Figure 1:**
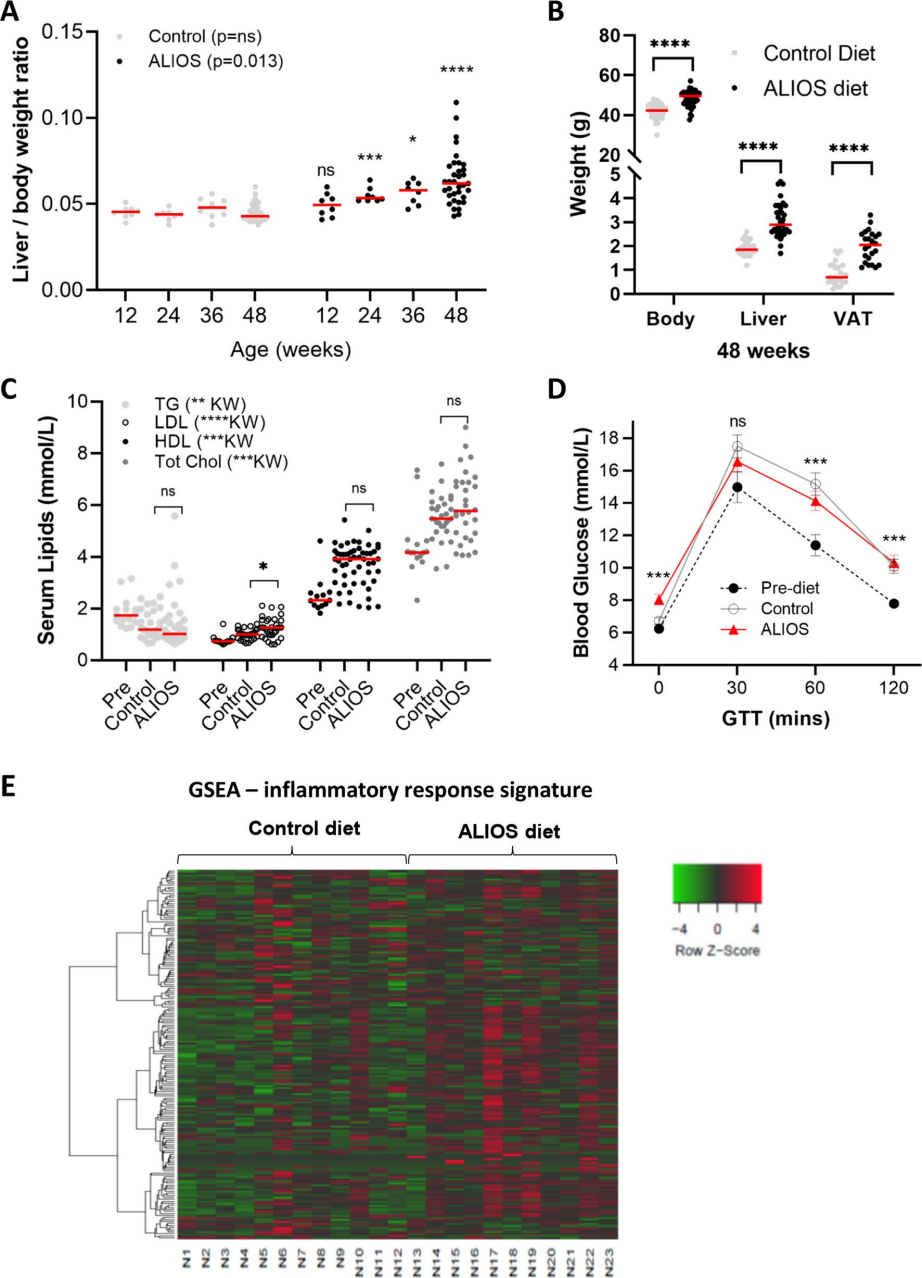
Aged C3H/He mice fed the ALIOS diet develop the metabolic syndrome. Liver/body weight ratio increased with age in ALIOS fed mice (**A**), with elevations in body liver and visceral adipose tissue (VAT) at 48 weeks (**B**). Lipid profiles of 48 weeks mice differed regardless of diet compared to pre diet levels (8 weeks), with low density lipoprotein levels (LDL) being higher in ALIOS fed mice at 48 weeks (**C**). Significant age associated changes during a glucose tolerance test (GTT) in both 48 weeks dietary groups relative to pre-dietary intervention (**D**). The fasting blood glucose was significantly elevated in ALIOS fed mice at 48 weeks (**D**). Data presented as mean ± S.E.M., Statistics using Kruskal Wallis or Mann–Whitney Tests, ns—not significant, *p < 0.05, **p < 0.01, ***p < 0.001, ****p < 0.0001. A heatmap created using heatmapper.ca^42^ and an inflammatory response signature including 182 genes shows enrichment in the non-tumour livers of ALIOS fed mice (N13-N23) compaired to controls (N1-N12), as well as heterogeneity within the dietary groups. GSEA—gene set enrichment analysis.

The ALIOS diet, representing popular ‘fast food’ meals, is associated with the development of NAFLD and 1-2mm liver tumours in C57Bl/6 mice at 1 year^14,18^. C3H/He mice are more susceptible to obesity and HCC. Activation of the Ras/Raf/MEK/ERK pathway is common in murine hepatocarcinogenesis regardless of the strain, with the liability of C3H/He attributed to an enhanced acquisition of spontaneous or induced Ha-ras oncogene mutation^19^. C3H/He mice were fed either ALIOS or control diet, with data summarised in Fig. 1 (F1) and supplementary Fig. 1 (SF1). ALIOS fed mice had significantly higher body weight (SF1A, F1B), liver weight (F1B, SF1B) and liver/body weight ratio (F1A) from as early as 12 weeks of age (4 weeks of diet), increasing stepwise at 24, 36 and 48 weeks of age. Visceral adipose tissue weight was also elevated (F1B). Age associated increases in serum lipids were similar in both groups of mice at 48 weeks relative to pre-diet at 8 weeks of age, although Serum LDL was higher (F1C). Serum alanine transaminase (ALT) was also elevated in ALIOS fed mice, in keeping with a greater degree of liver injury (SF1C). Neither dietary group developed diabetes, as defined by a GTT performed at 48 weeks compared to 8 weeks, but both dietary groups had elevated glucose levels at 2 hours, in keeping with age related impaired glucose tolerance (F1D). In addition, the ALIOS fed group developed impaired fasting blood glucose (FBG) (8.05 ± 0.32 versus 6.72 ± 0.19; p = 0.001) (F1D), in keeping with ALIOS diet associated insulin resistance. The expression of inflammatory markers, tumour necrosis factor alpha (Tnf*α*) and inducible nitric oxide synthase (iNos), was up-regulated in ALIOS diet liver tissues compared to control diet mice (SF1D), in keeping with a higher level of inflammation. A heatmap including a 182 gene inflammatory response signature^20^ is shown in F1E, showing that the majority of ALIOS fed mice had a higher level of inflammation, but also a degree of heterogeneity within the dietary groups.

**Figure 2:**
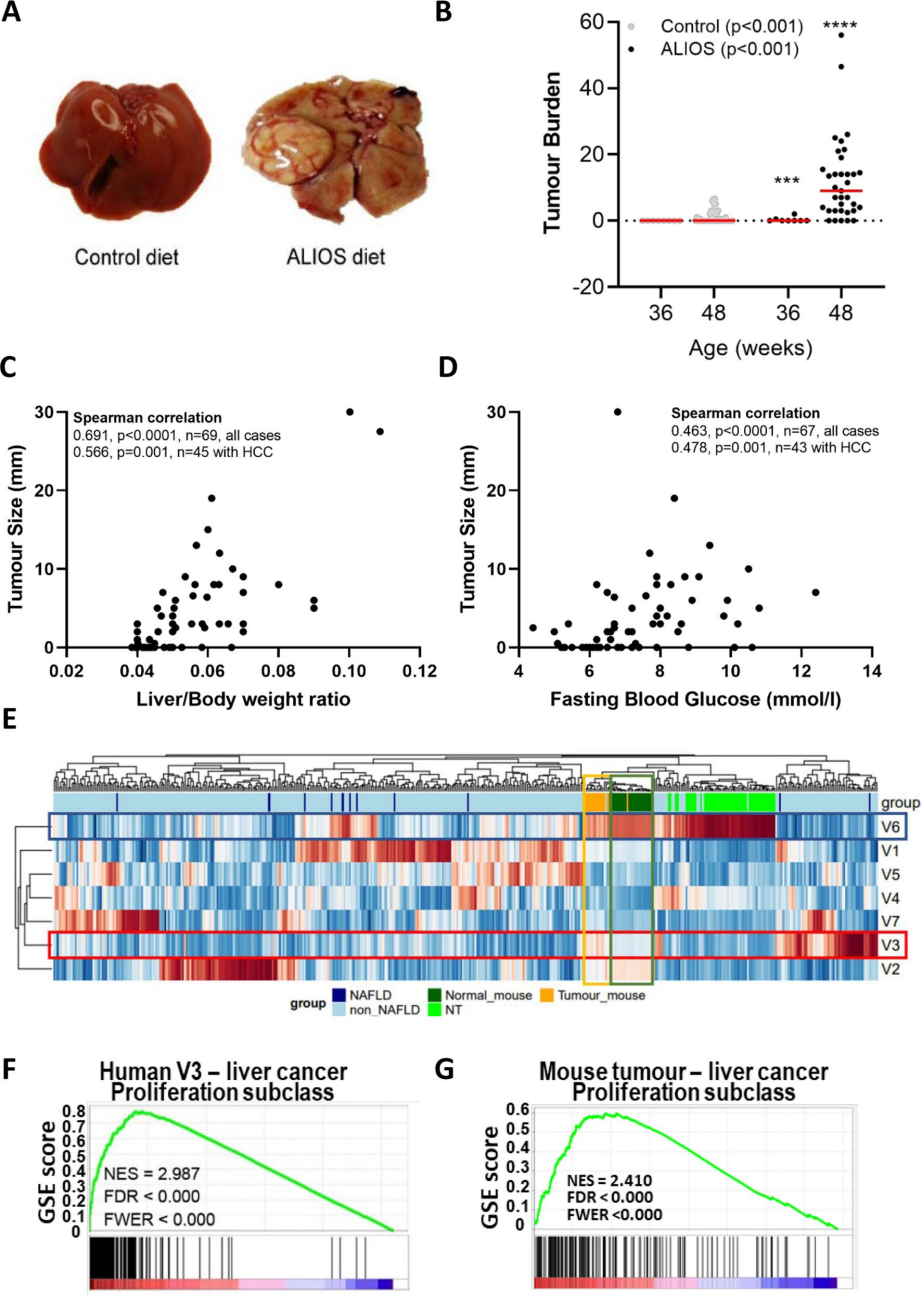
Aged C3H/He mice fed the ALIOS diet develop HCC. Livers of ALIOS fed mice at 48 weeks appeared steatotic with visible tumours (**A**), with elevated tumour burden (**B**) relative to control. Tumour size increased in association with liver/weight ratio (C) and fasting blood glucose (**D**). The transcriptome of mouse tumours overlapped with a distinct human subclass of HCC, comparing RNA-seq data (**E**–**F**). In the heat map of Non-negative Matrix Factorization (NMF) (**E**),tissue type is colour coded (top row). Red and blue represent higher and lower gene expression. The human non-tumour (NT) metagene is marked V6 (blue rectangle), with tumour (T) metagenes defined as V1–V5 and V7. A red rectangle marks the V3 human metagene. Mouse T and NT transcriptomes are shown by orange and green rectangles. A proliferative subclass was identified by the Gene set enrichment analysis (GSEA) score of top enriched dataset in human V3 (F), and the mouse T versus NT differentially expressed genes (G). Data in (B) presented as mean ± S.E.M., compared with Mann Whitney test.

Tumour data are summarized in Fig. 2 (F2). Livers of ALIOS fed C3H/He mice appeared pale and macroscopically fatty, with spontaneous visible tumours being common at 48 weeks (F2A). Tumours were present in 15/33 control mice and in 30/35 (ALIOS fed mice (p<0.0001; Pearson Chi Square), with the number and size also being greater in ALIOS fed mice (F2B, SF1E). In mice culled at 36 weeks, macroscopic HCC were present at a lesser frequency (0/8 control diet, 2/8 ALIOS diet). Notably, body weight, liver weight and the liver/body weight ratio (F2C) were strongly associated with tumour development, tumour number and the size of the largest tumour (Table 1). FBG at 48 weeks (GTT2 0 minutes) was also highly significantly associated with tumour numbers and size (Table 1, F2D). There was a significant but weaker association with serum LDL, although no associations with elevated serum ALT (Table 1).

**Table 1:** Gross phenotype features associated with tumours. Means ± standard errors are shown for continuous data. Differences between continuous non-parametric were assessed with Mann–Whitney Tests. Key: BW—body weight; BG—blood glucose; GGT—glucose tolerance test; AUC—area under curve; HDL—high density lipoprotein; LDL—low density lipoprotein.

	Tumour development			Tumour number		Largest tumour	
	No	Yes	p value	Corr	p value	Corr	p value
Number	23	45		68		45	
Diet (control/ALIOS)	18/5	15/30	< 0.001	0.583	< 0.001	0.547	< 0.001
Liver weight (g)	1.94 ± 0.097	2.82 ± 0.12	< 0.001	0.732	< 0.001	0.553	< 0.001
Body weight (g)	42.56 ± 1.05	46.83 ± 0.65	0.002	0.491	< 0.001	0.168	0.271
Liver/BW ratio	0.045 ± 0.001	0.060 ± 0.002	< 0.001	0.681	< 0.001	0.566	< 0.001
VAT weight (g)	1.15 ± 0.15	1.67 ± 0.16	0.030	0.343	0.018	0.071	0.725
**BG (mmol/l) glucose tolerance test at 48 weeks (GGT2)**							
BG GGT2 0 min	6.76 ± 0.32	7.72 ± 0.25	0.012	0.403	0.001	0.478	0.001
BG GGT2 30 min	17.13 ± 0.82	17.08 ± 0.61	ns	-0.071	ns	-0.251	ns
BG GGT2 60 min	14.45 ± 0.93	14.90 ± 0.49	ns	0.048	ns	-0.275	ns
BG GGT2 120 min	9.79 ± 0.55	10.53 ± 0.39	ns	0.098	ns	-0.081	ns
GGT2 AUC	1559.5 ± 70.6	1614.8 ± 46.1	ns	0.046	ns	-0.208	ns
**Lipid profile and alanine transaminase (ALT) tests at 48 weeks**							
Cholesterol (mmol/l)	5.24 ± 0.21	5.84 ± 0.23	0.091	0.163	ns	0.319	ns
HDL cholesterol (mmol/l)	3.46 ± 0.17	3.53 ± 0.20	ns	0.016	ns	0.106	ns
LDL cholesterol (mmol/l)	0.91 ± 0.05	1.14 ± 0.07	0.021	0.317	0.046	0.295	ns
Triglycerides	1.27 ± 0.14	1.15 ± 0.17	ns	0.121	ns	0.002	ns
ALT (IU/L)	42.71 ± 12.84	44.28 ± 7.62	ns	0.232	ns	0.222	ns

Pilot and follow up study, 48 weeks, n = 69.

Expression of tumour markers Afp, Gpc3 and Nope were significantly higher in the tumours versus the adjacent non-tumour tissues, this in keeping with the tumours being HCC (SF1F). In fourteen tumours there was sufficient tissues for RNA extraction and RNA-seq analysis. These were from one control diet and 13 ALIOS fed mice. Unsupervised clustering analysis distinguished the tumour tissues from the non-tumour tissues (SF2). Mutations in the Ras oncogene were highly prevalent in tumour tissues only, including tumours from the control diet animal and 9/13 ALIOS fed animals. Data can be accessed in GSE137407. To explore overlap between the tumours in our model and human HCC, the mouse transcriptomic data was compared to publicly available human data from The Cancer Genome Atlas Liver Hepatocellular Carcinoma (TCGA-LIHC) project. Unsupervised class discovery of 374 liver cancer and 50 normal tissue samples in the dataset by NMF-metagene and K-means clustering identified a single non-tumour (V6) and 6 tumour metagenes (V1-5, V7), similar to the main biological subtypes previously reported by clustering of this dataset^21^. By projecting metagenes onto the mouse RNA-seq dataset, non-tumour mouse gene expression was enriched in the human non-tumour V6 metagene, indicative of a comparable liver transcriptomic signature across the two species. The mouse tumour gene expression was enriched with the human V3 HCC metagene (F2E). GSEA analysis of the human metagene V3 DE gene list revealed a signature characterised by enhanced proliferation, high serum AFP and chromosomal aberrations (F2F). This gene signature was also enriched in the C3H/He mouse tumour transcriptomic data (F2G).

### Histological characterisation of NAFLD and HCC

**Figure 3:**
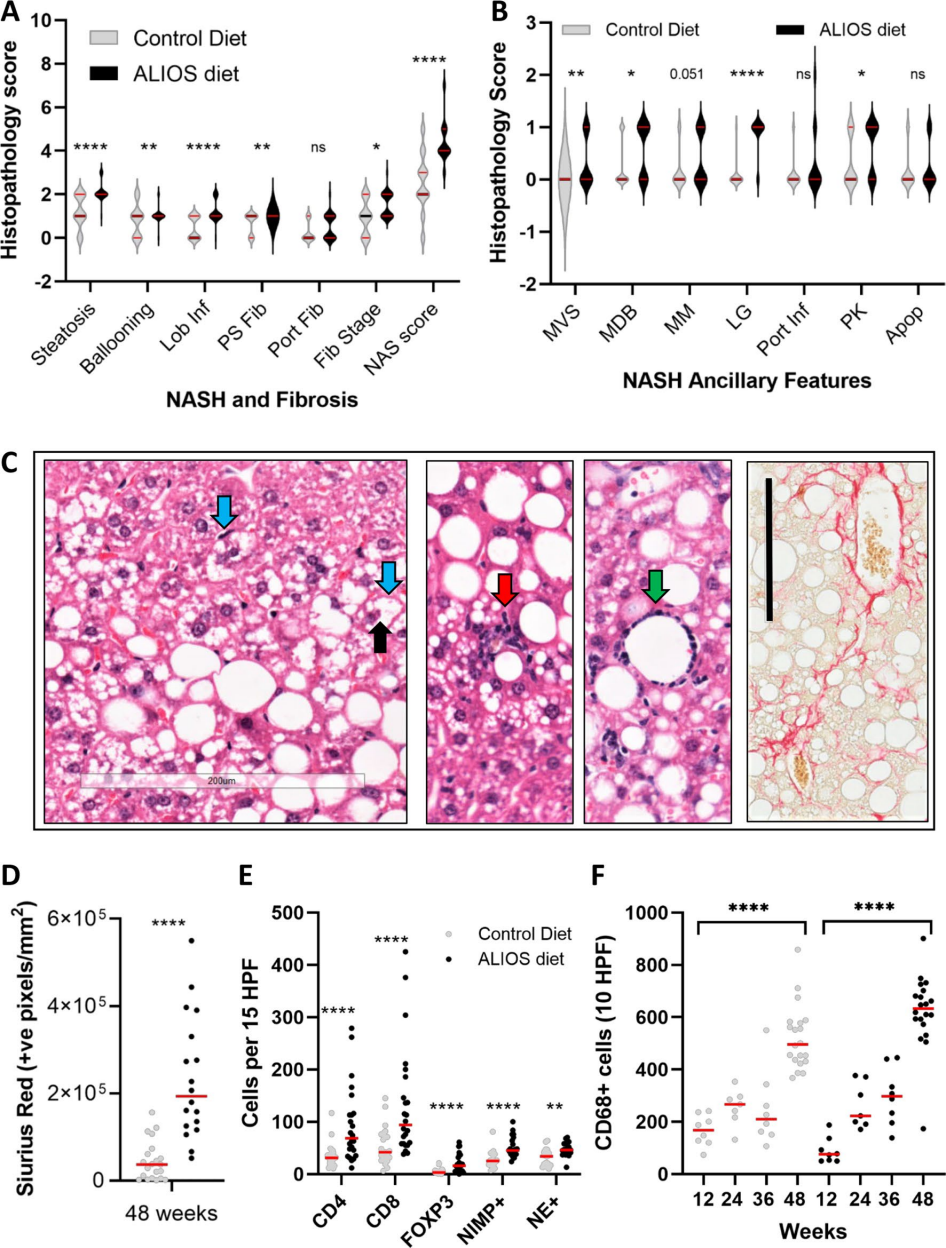
DINAH model histopathology features of NASH. In the livers of 48 weeks control diet C3H/He mice, mild to moderate NAFLD was common, although expert histological assessment confirmed ALIOS induced features of more severe disease, with significantly increased steatosis, lobular inflammation (Lob Inf) and fibrosis (**A**), as well as microvesiular steatosis (MVS), Mallory Denk Bodies (MDB), lipogranulomas (LG) and pigmented Kupffer cells (KP). Arrows in (**C**) depict lobular inflammation (red), ballooned hepatocytes (blue), Mallory–Denk bodies (black) and macrophages around a lipid droplet—a lipogranuloma (green). Sirius Red (**C**, far right) positive pixels captured by Aperio Image analysis confirmed significant elevations in pericellular fibrosis in ALIOS fed mice at 48 weeks (**D**), with an example in (**C**, far right). There were significant elevations in CD4 + , CD8 + and FOXP3 + T lymphocytes, as well as NIMP + and neutrophil elastase + neutrophils (**E**). Quantification of CD68 + macrophages confirmed a step wise increase from 12 to 48 weeks of age, more marked in ALIOS fed mice (**F**). Statistical differences were determined by Chi Square or Mann–Whitney Tests (ns = not significant, ** p < 0.01, *** p < 0.001, **** p < 0.0001. Additional Key: PS—perisinusoidal, Fib fibrosis, MM—megamitochndria, Port—portal, Apop -apoptosis.

NAFLD histological features in control versus ALIOS diet fed mice are summarised in Fig. 3 (F3). NAFLD was defined histologically by the presence of steatosis in > 5% of hepatocytes. Steatosis was evident in the majority of the mice, regardless of control or ALIOS diet, by 24 weeks of age. The severity of steatosis increased with age and at 48 weeks was significantly more pronounced in ALIOS diet fed mice compared to control mice, as was lobular inflammation (F3A). As a marker of a chronic injury wound healing response, fibrosis was common in both dietary groups of mice, with elevated fibrosis stage 1–2 in ALIOS fed mice, versus 0–1 in control mice (F3A). Peri-sinusoidal fibrosis was more common at 48 weeks in ALIOS (100%, 24/24) compared to control diet mice (69.5%, 16/23) (F3A), confirmed by semi-quantitative image analysis of Sirius Red stain (F3D). Ballooning was present in 95% of ALIOS versus 65% of control diet mice, with the combined NAFLD activity score (NAS score)^22^ significantly higher (F3A). Representative histopathology features are shown in F3C. IHC characterisation of the immune infiltrate confirmed increases in CD8 (cytotoxic), CD4 (helper) and FOXP3 (regulatory) T cells, as well as neutrophils (F3E). CD4 and CD8 T cells were the most prevalent, but the elevation induced by diet was most notable for FOXP3 T cells (4 fold), which were scant in control diet livers. Ancillary features of NASH, Mallory-Denk bodies and megamitochondria, were also present. NAFLD histological features such as microvesicular steatosis, pigmented Kupffer cells and lipogranulomas^22^ were rare at 12-36 weeks, but were much more common at 48 weeks and in ALIOS diet fed mice (F3B). This was most striking for lipogranulomas, detected in just 1/23 control and 21/24 ALIOS diet fed mice (p < 0.001). A lipogranuloma consists of a steatotic hepatocyte or fat droplet, surrounded by mononuclear cells and macrophages and an occasional eosinophil (F3C). Counts of CD68+ macrophages confirmed these were elevated in both dietary groups of animals, more so in those fed ALIOS diet and at an earlier age (36weeks) compared to control diet mice (F3F).

**Figure 4:**
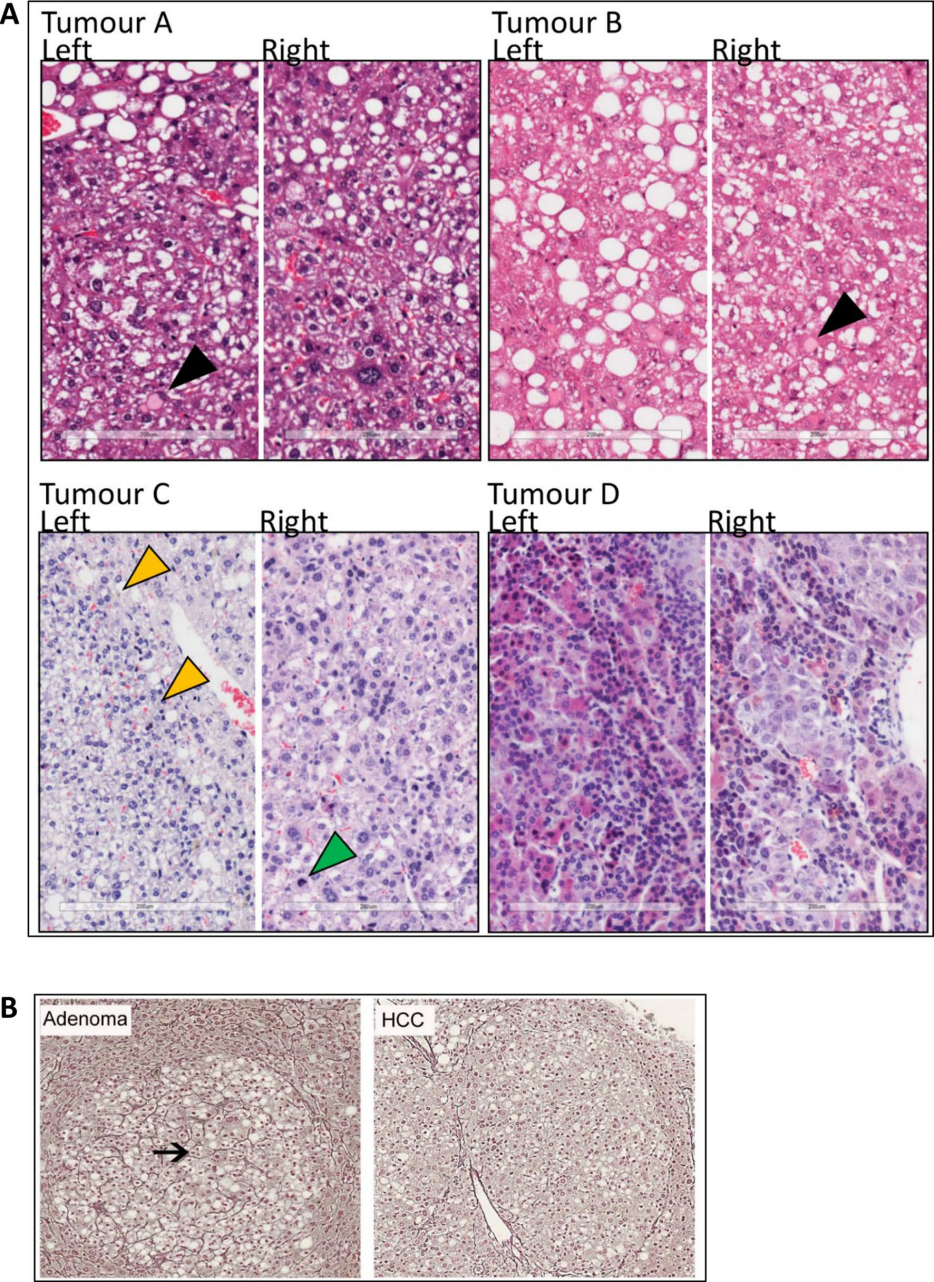
DINAH model histopathology features of HCC. Heamatoxylin and Eosin immunohistochemistry from 4 tumours is shown (**A**). Tumours A and B are hepatocellular neoplasms showing steatosis, focally increased nuclear-cytoplasmic ratio, nuclear atypia and eosinophilic cytoplasmic inclusions (black triangles) in keeping with HCC. In tumour C, yellow triangles (left) highlight bile production, with green triangle (right) showing a mitotic figure also indicative of HCC. Silver stain shows preservation of reticulin between liver cell plates in an adenoma (black arrow), with loss of reticulin and thickened liver cell plates in a tumour classed HCC (**B**).

As reported above, 45/68 mice had tumours that were classed as HCC. Histological characterisation of tumours, presented in Fig. 4 (F4) revealed common steatohepatitic features. Nuclear atypia, eosinophilic inclusions, frequent mitoses and thickened (> 2) tumour cell plates, confirmed by reticulin staining, distinguished steatohepatitic hepatocellular carcinomas (SH-HCC) from benign adenomas (F4A, F4B).

### Histological features associated with NAFLD-HCC development

Histological associations with NAFLD-HCC development in C3H/He mice at 48 weeks (15/33 control diet; 30/35 ALIOS diet) are summarised in Table 2. Considering NASH features, there was no association with the presence of hepatocyte ballooning, although correlations with the severity of steatosis, lobular inflammation and the NAS score were highly significant. There were weaker associations with the presence of fibrosis. Ancillary features of NAFLD were also considered. Of these, the presence of lipogranulomas was most strikingly and significantly associated with NAFLD-HCC development. The presence of Mallory–Denk bodies was the feature most significantly associated with tumour size. Of the T cell subsets, the CD4 T cells were the most strongly and significantly associated with the development of tumours, the number of tumours and tumour size.

**Table 2:** Histological features associated with tumours. Differences between continuous non-parametric were assessed with Mann–Whitney Tests. Differences between categorical datasets were assessed with Chi Square Exact tests. Immune cell counts are shown for 15 high power fields (HPF) for T cells and neutrophils, with the mean of 10 HPF shown for macrophages. Macrophage data are on a subset of 23 cases. Key: MV—microvesicular; MDB—Mallory Denk bodies; LI—lobular inflammation; PI—portal inflammation; MM—megamitochnodria; PK—pigmented Kupffer cells; PS—peri-sinusoidal.

	Tumour development			Tumour number		Largest tumour	
	No	Yes	p value	Corr	p value	Corr	p value
Number	20	27		47		27	
Steatosis (0/1/2/3)	4/7/8/1	0/4/20/3	0.009	0.429	0.003	0.254	ns
MV steatosis (0/1)	19/1	20/7	0.059	0.382	0.008	0.038	ns
Ballooning (0/1/2)	5/12/3	4/20/3	ns	0.058	ns	0.109	ns
MDB (0/1)	14/6	13/13	ns	0.305	0.039	0.581	0.002
Lipogranuloma (0/1)	15/5	9/17	0.007	0.481	0.001	0.438	0.025
LI (0/1/2)	9/10/1	4/18/5	0.057	0.421	0.003	0.349	ns
PI (0/1/2)	18/1/1	21/5/1	ns	0.276	ns	0.189	ns
MM (0/1)	16/4	17/9	ns	0.031	ns	-0.335	ns
Apoptotic cells (0/1)	15/5	22/4	ns	-0.114	ns	0.356	ns
PK cells (0/1)	13/7	11/15	ns	0.280	ns	0.302	ns
PS fibrosis score (0/1)	6/14	1/26	0.012	0.361	0.013	0.038	ns
Fibrosis stage (0/1/2/3)	6/8/6	1/14/11/1	0.064	0.355	0.014	0.046	ns
NAS (0/1/2/3/4/5/5+)	2/2/4/6/4/1/1	0/0/4/2/13/7/1	0.012	0.465	0.001	0.307	ns
Peri-portal γH2AX	2.00 ± 0.65	4.03 ± 0.82	0.017	0.402	0.006	0.356	0.015
Peri-portal Ki67	1.96 ± 0.45	3.17 ± 0.47	0.012	0.550	0.007	0.542	0.007
CD4 T cells	46.75 ± 13.41	77.15 ± 11.37	0.004	0.428	0.003	0.512	< 0.001
CD8 T cells	68.50 ± 14.96	112.8 ± 18.76	0.023	0.335	0.021	0.369	0.011
Foxp3 T cells	8.35 ± 2.68	16.33 ± 3.20	0.041	0.301	0.040	0.380	0.009
NIMP + neutrophils	31.50 ± 4.11	46.19 ± 4.29	0.033	0.337	0.031	0.347	0.026
Elastase + neutrophils	34.47 ± 4.07	43.29 ± 3.43	ns	0.262	ns	0.397	0.012
CD44 + macrophages*	21.99 ± 6.33	72.03 ± 12.41	0.009	0.602	0.003	0.456	0.033
CD68 + macrophages*	67.78 ± 5.08	88.17 ± 5.10	0.023	0.647	0.002	0.449	0.041
F4/80 + macrophages*	53.8 ± 7.1	104.8 ± 20.8	0.071	0.442	0.039	0.363	0.097

Follow-up study at 48 weeks, n = 47; *subset of cases used for RNA-Seq (n = 23).

### C3H/He NAFLD-HCC arises in a proliferative steatotic environment with persistent inflammation

**Figure 5:**
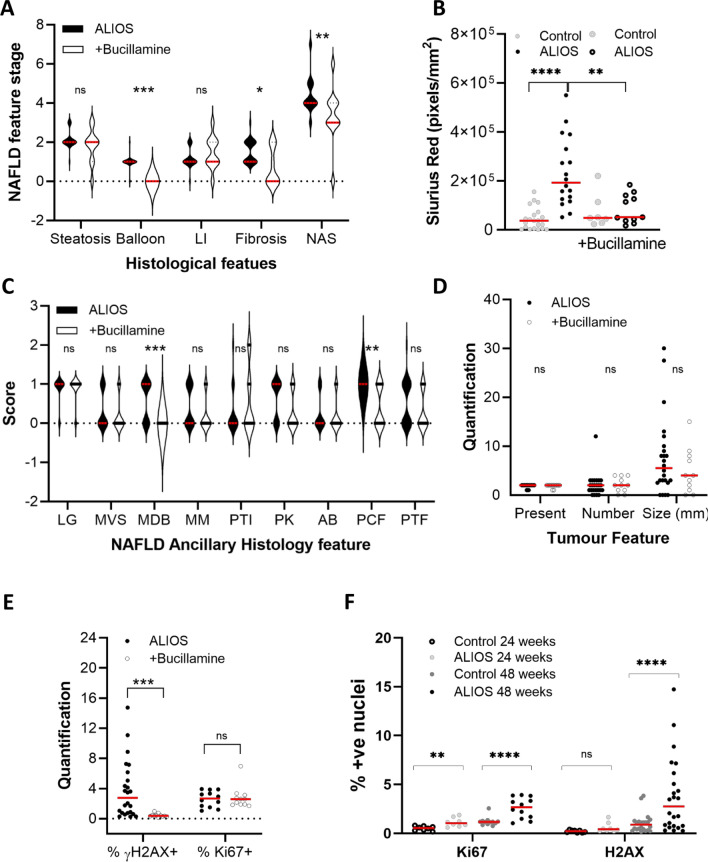
The impact of bucillamine on NASH, DNA damage, cellular proliferation and HCC. Dietary supplementation with bucillamine in ALIOS fed C3H/He mice had no impact on steatosis or lobular inflammation, although a cardinal feature of NASH—hepatocyte ballooning—was abrogated, with a corresponding reduction in the NAS score, alongside a significant reduction in fibrosis stage (**A**) and Sirius Red quantification (**B**). Of the ancillary features of NASH, Mallory-Denk bodies (MDB) were reduced, as was pericellular fibrosis (PCF), with no changes in the presence of lipogranulomas (LG), microvesicular steatosis (MVS), megamitochondria (MM), portal tract inflammation (PTI), pigmented Kupffer (PK) cells, apoptotic bodies (AB) or portal tract fibrosis (PTF) (**C**). Despite reduced severity of NASH and fibrosis, there was no impact tumour development or size (**C**). DNA damage H2AX foci were diminished, although the hepatocyte proliferation biomarker Ki67 was not (**E**). Quantification at 24 weeks of age confirmed a significant increase in Ki67 assessed proliferation in ALIOS fed mice, further increased at 48 weeks. Induction of H2AX was more dramatic in ALIOS fed mice, but only at 48 weeks. Categorical data statistical differences determined by Chi Square tests; scale data by Mann Whitney Tests (ns-not significant, * p < 0.05, ** p < 0.01, *** p < 0.001).

Parallel groups of mice on ALIOS and control diets received bucillamine a cysteine derivative used first line in Japan as a disease modifying drug to treat rheumatoid arthritis^23,24^. Bucillamine behaves as a thiol anti oxidant reducing reperfusion injury in pre clinical models of liver transplantation, replenishing glutathione and promoting defences against oxidant injury in both normal and steatotic livers^25^. Data are summarised in Figure 5 (F5), SF3 and Supplementary Results Table 2. There was a dramatic reduction in the presence and severity of hepatocyte ballooning and consequently the NAS score in ALIOS diet fed mice (F5A). Encouragingly, dietary bucillamine also reduced the ALIOS diet associated hepatic fibrosis stage at 48 weeks (F5A), as well as pericellular fibrosis (F5A-C). Despite the positive impact on the severity of NAFLD, bucillamine treatment reduced neither numbers nor size of HCC at 48 weeks (F5D). We explored the impact of bucillamine on other features, including those associated with elevated tumour incidence. Bucillamine had minimal impact on body weight, liver weight or blood glucose (SF3A). Regarding liver histopathology, there was also little impact on the steatosis grade or lobular inflammation score in the ALIOS fed mice, with also no impact on the presence of lipogranulomas (F5C). Of the other ancillary features of NAFLD, there was a highly significant reduction in Mallory-Denk bodies indicative of reduced hepatocyte injury. Strikingly, we noted a substantial reduction in the percentage of *γ*-H2AX+ nuclei, indicating a degree of bucillamine associated protection from DNA damage (F5E, SF3B). In contrast, Ki67 assessed hepatocyte proliferation was not impacted by bucillamine (F5F, SF3B). While *γ*–H2AX foci were dramatically elevated in ALIOS fed mice, this was most notable in aged mice at 48 weeks (F5F). Ki67 on the other hand, was elevated in younger ALIOS fed mice, at 24 weeks, with a further increase at 48 weeks (F5F). Of note, Ki67 was significantly associated with FBG, liver weight and HCC development at 48 weeks.

In NAFLD, fibrosis is a critical prognostic factor in patients^5^, although patients without it can develop HCC. These data reveal that in C3H/He mice, a chronic proliferative environment is associated with steatosis and an elevated FBG, with the acquisition of lipogranulomas and mild lobular inflammation, sufficient to promote HCC development, even when levels of cellular and DNA damage were low and cirrhosis absent.

### Transcriptomic studies—immune signatures associated with NAFLD-HCC development

**Figure 6:**
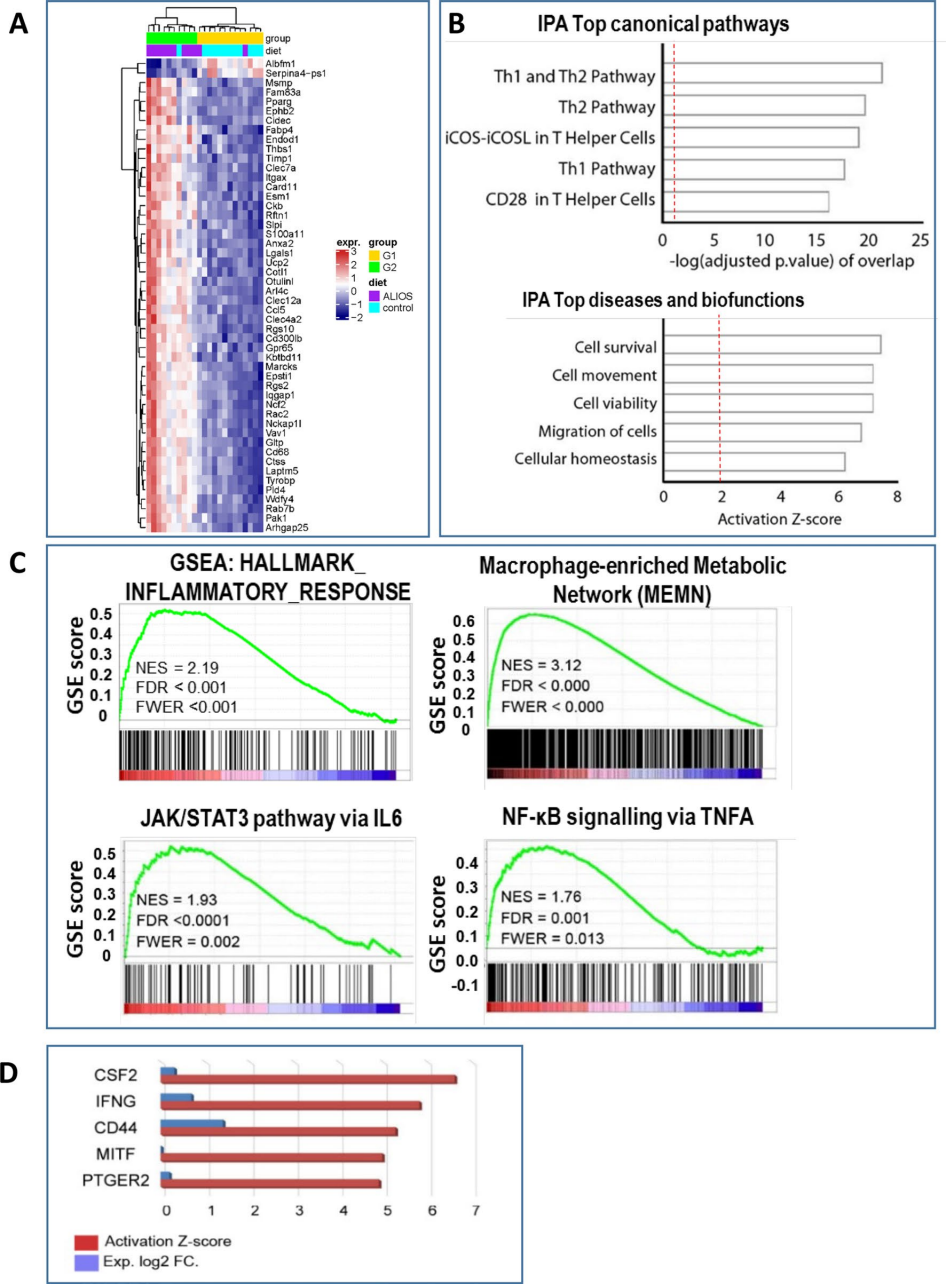
DINAH model transcriptome in NAFLD non-tumour identifies inflammatory pathways, macrophages and CD44 associated with HCC development. The RNA-seq transcriptome heat map, generated after unsupervised clustering analysis of non-tumour (NT) C3H/He, compares the two distinct groups identified (**A**), where G2 distinguished mice bearing greater number and size of tumours. Ingenuity pathway analysis (IPA) of the differentially expressed (DE) genes identified top canonical pathways ranked according to the-log adjusted p-value, or top diseases and bio-functions activation score (**B**). Gene Set Enrichment Analysis (GSEA) highlighted and inflammatory response signatures, the Macrophage enriched metabolic network, NF-*κ*B and JAK/STAT3 pathways (**C**) as enriched in G2 mice. Considering the top IPA upstream regulators of the non-tumour G2-G1 transcriptomic profile, CD44 was ranked third among activated regulators (red) and was the one with the highest differential expression (DE) (blue) (**D**). Key: NES—normalised enrichment score; FDR—false discovery rate adjusted value; FWER—family-wise error rate.

To investigate the candidate contributory pathways and upstream regulators leading to the development of HCC in C3H/He mice, RNA-sequencing (RNA-seq) was performed on 23 non-tumour liver tissues with a range of histological severities of NAFLD, with data summarised in Fig. 6 (F6). Unsupervised clustering analysis of the RNA-seq data identified two distinct groups (G1 and G2). Differences between dietary, gross and histological features of G1 and G2 are summarised in SF4, ranked according to significance. The G2 cluster mice were more commonly on the ALIOS diet, with higher liver and body weight, together with higher steatosis grade, fibrosis stage, higher NAS score and more frequent lipogranulomas compared to the mice in the G1 cluster. Notably, G2 mice developed bigger tumours and had a higher tumour burden compared to mice in the G1 subgroup. 3,900 genes were differentially expressed (DE) between the G2 and G1 groups with an adjusted p-value of less than 0.05. The top 100 DE genes are shown in Supplementary Results Table 3 and the top 50 in Fig. 6A. Ingenuity pathway analysis (IPA) of the DE gene list identified an activation of Th1 and Th2 pathways ranking amongst the top enriched canonical pathways. IPA also identified cell survival and viability, together with cellular movement and infiltration, as the top activated bio-functions (F6B), in keeping with the elevation of Ki67 as a biomarker of a ‘proliferative’ histological phenotype observed in C3H/He mice developing HCC. Gene set enrichment analysis (GSEA) of the same list recognised an inflammatory response signature (F6C), with a macrophage signature as the top enriched canonical pathway (F6C). Activation of the NF-*κ*B and STAT3 inflammatory pathways was also evident (F6C), with activation of these two pathways in the non-tumour liver tissues of HCC patients previously reported to be associated with higher tumour recurrence^26^.

#### Enrichment of CD44+ve macrophages, in association with T2DM and HCC

IPA analysis for the top upstream regulators of gene expression in the non-tumour tissues G2 cluster (F6) identified CD44 as the most differentially expressed (F6D). CD44 was also present in the gene lists from both the IPA defined Top Diseases Pathway and the GSEA defined Macrophage Enriched metabolic Network, with a role in recruitment of T lymphocytes implicated.

**Figure 7:**
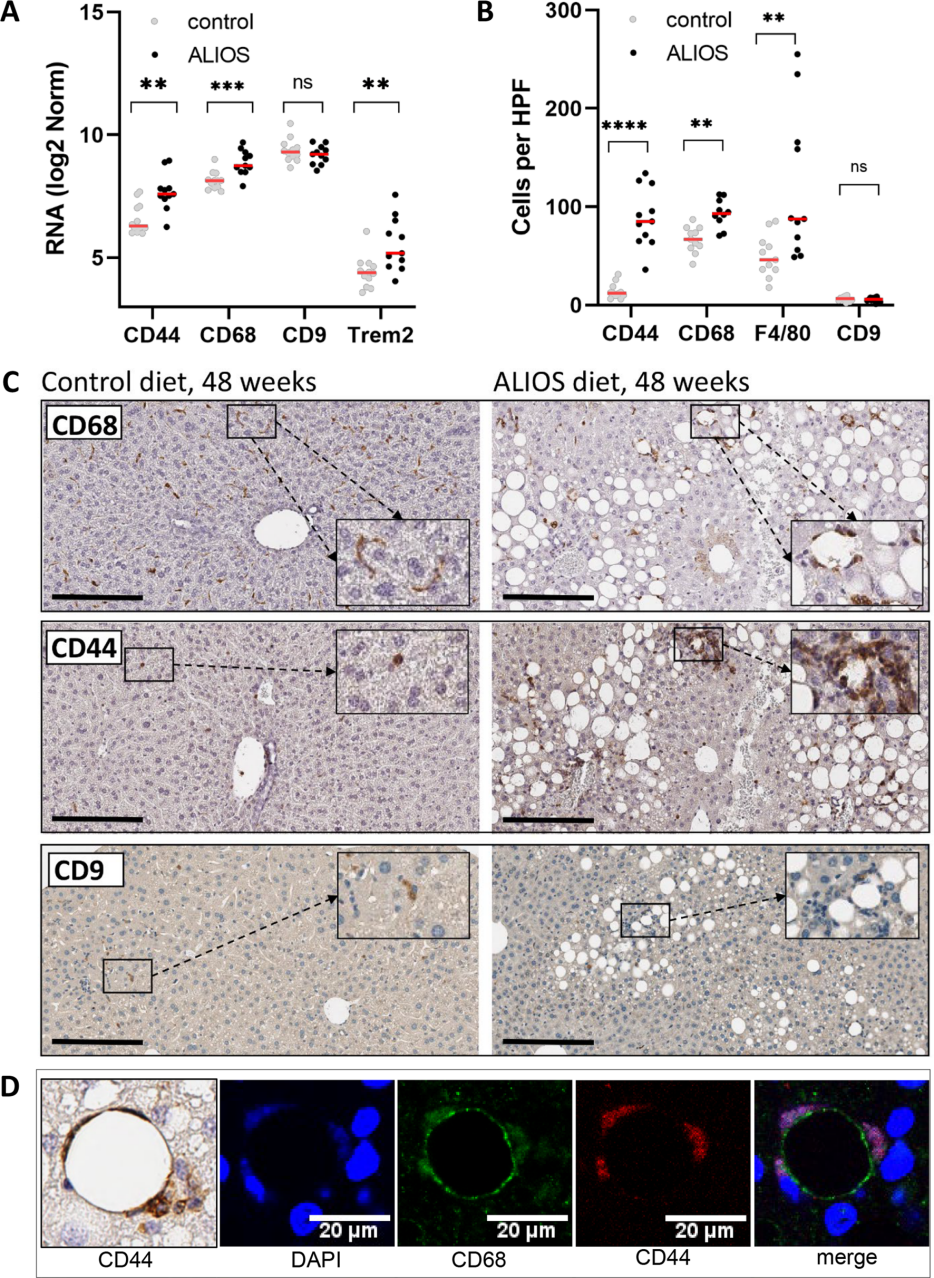
Elevations in CD44 + macrophages in aged, fatty non-tumour liver. Messenger RNA levels in RNA-Seq studies of whole liver tissues at 48 weeks confirmed elevations in CD44, CD68 and Trem2 in ALIOS fed animals, while CD9—present at relatively high levels in control and 48 week ALIOS fed mice—was unchanged (**A**). Counts of positive protein expressing cells detected by IHC followed a similar pattern, although Trem2—with the lowest transcript levels—was not detected in FFPE tissues (not shown). The most striking change was for CD44 + cells, which were scant in controls but prominent in ALIOS fed mice—notably in lipogranulomas (**C**), with dual staining confirming co-expression of CD68 and CD44 (**D**). Counts are shown as numbers per high power field (HPF). ns—not significant; * < 0.05;**p < 0.01; ***p < 0.001; ****p < 0.0001 Mann–Whitney Tests.

CD44 transcript expression in whole liver was explored in the mouse RNA-Seq dataset Fig. 7 (F7), alongside the classical macrophage marker CD68 and two recently identified markers, CD9 and Trem2, associated with NAFLD progression^27–29^. Messenger RNA transcript levels of both CD68 and CD9 were relatively high in 48 weeks mice, with CD68 further increased in mice fed ALIOS diet (F7A). Levels of CD44 were lower in control mice, but rose quite dramatically in ALIOS fed (F7A). Trem2 levels also increased in ALIOS fed mice, but transcript levels in whole liver were low (F7A). These findings were supported at the cellular protein levels by IHC in the mouse tissues (Control diet n = 12, ALIOS diet n = 11), as summarised in (F7B-D). CD44 expression was predominantly in macrophages, characterised by their location within sinusoids and lipogranulomas, but also by association with CD68 and F4/80 expressing cells and dual immunofluoresecent labelling confirming co-expression of CD44 in CD68 expressing macrophages (F7C-D, SF5A-B). Comparing macrophage numbers, resident CD68 and F4/80 positive cells were relatively common in control mice at 48 weeks of age, rising further in ALIOS fed mice. The numbers of CD44+ cells were scant in control diet mice, with a dramatic increase in ALIOS fed mice (88.79±9.1 versus 14.32±2.3 per high power field respectively, p < 0.0001), reaching similar levels to CD68 and F4/80+ positive cells (F7B). Despite high transcript levels in whole liver, CD9+ macrophages were relatively scant in C3H/He livers at 48 weeks, with no significant change induced by ALIOS diet (F7A-B). Trem2 was not discerned by IHC in our FFPE liver tissues from C3H/He mice (data not shown).The number of CD44+ macrophages was strongly associated with the development of tumours and tumour number (Table 2). CD44+ cells, rather than CD68+ or F4/80+ cells, also correlated strongly and significantly with CD4, FOXP3 and CD8 T cells (Spearman correlations 0.636, p = 0.001; 0.548, p = 0.008; 0.636, p = 0.001 respectively). Dietary supplementation with bucillamine had no impact on the presence or absence of liopgranulomas in ALIOS fed mice at 48 weeks (F5C), nor numbers of CD44+ macrophages (SF5C-D).

**Figure 8:**
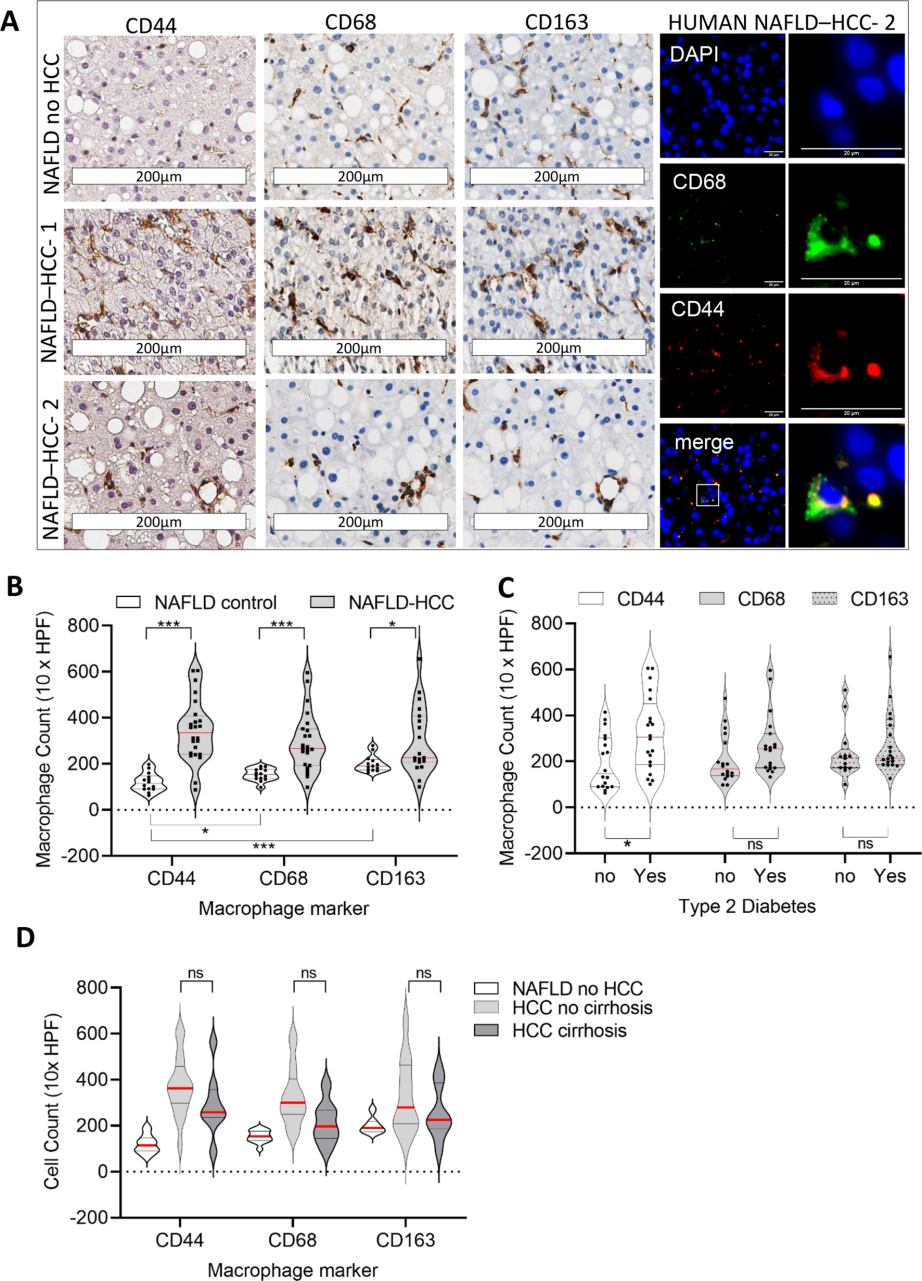
Elevations in CD44 + macrophages in non-tumour Human liver, in association with steatosis, insulin resistance and HCC. In Human NAFLD, CD44 + cells were scant compared to CD68 + or CD163 + macrophages (**A**–top row). In contrast, CD44 + cells increased in association with sinusoidal CD68 + or CD163 + cells in the NT liver of patients with HCC (A-middle row), as well as in lipogranulomas (**C**–bottom row). Dual CD44 and CD68 stain confirmed their co-expression in macrophages in Human NAFLD (A-far column). CD44 + cell counts in NAFLD without HCC were fewer than CD68 + (p < 0.05) or CD163 + (p < 0.01) cells, with a more dramatic increase in NT liver of patients with NAFLD-HCC, compared to increases in CD68 and CD163 (**B**). For CD44 + macrophages only, there was a significant increase in NT tissues of patients with Type 2 diabetes (**D**). NAFLD-HCC associated elevations in macrophages regardless of the marker (CD44, CD68, CD163) were present in both the presence or absence of cirrhosis. Counts are shown as numbers in 10 high power fields (HPF); ns-not significant; * < 0.05;**p < 0.01; ***p < 0.001; Mann–Whitney Tests.

Data from FFPE Human (NAFLD-no-HCC n = 15; NAFLD-HCC n = 27) non-tumour liver biopsy tissues is summarized in Fig. 8 (F8). The distribution of CD44+ cells in NAFLD cases with and without HCC is represented in F8A, showing similar distribution in sinusoids and lipogranuolams to CD68+ and CD163+ cells and co-expression with CD68 confirmed by dual labelling immunofluorescence. In human NAFLD cases, although there were strong correlations between CD44+ and CD68+ (Spearman 0.735, p < 0.001) and CD163+ (Spearman 0.695, p < 0.001) cells, the level of CD44 was significantly lower in control NAFLD cases without HCC (F8B) (p < 0.001 relative to CD163 and p < 0.05 relative to CD68). Consequently, the increase seen in CD44+ macrophages in human cases with NAFLD-HCC was much more dramatic (F8B). The increases in each of the macrophage subtypes seen in non-tumour liver in association with NAFLD-HCC were independent of the presence or absence of cirrhosis (F8D), although the increase in CD44+ macrophages was significantly associated with the presence of T2DM (F8C). There was no significant T2DM association with CD68+ or CD163+ macrophages (F8C).

## Discussion

NAFLD-HCC patients are often older, with metabolic syndrome associated comorbidities, diagnosed at a more advanced stage of disease owing to lack of or ineffective HCC surveillance^30,31^. To develop preventive and therapeutic approaches for this target population, there is a need to better understand the pathogenesis of HCC in these contexts.

A number of animal models have been used to explore hepatocarcinogenesis in NAFLD^5^, with some of those involving dietary manipulations as described in the introduction. While caution is advised interpreting data from any model in isolation, particularly given the transcription profiles of tumours tend not to recapitulate human disease^32^, significant advances in our understanding of tumour development in fatty livers have been made. It is known that increases in circulating fatty acids lead to steatosis, with intrahepatic lymphocytes and inflammatory macrophages, along with inflammatory cytokines, leading to chronic cellular injury, DNA damage and hepatocyte death^5^. NAFLD progression to NASH and fibrosis, contributes to the increased risk of HCC^5^. Our aim was to compliment these studies by exploring features not necessarily dependent on NASH progression to fibrosis—given up to 50% of humans with NAFLD-HCC develop it in the absence of advanced fibrosis or cirrhosis. As the majority of dietary studies thus far have used C57Bl/6 mice, which do not develop the metabolic syndrome with age and require additional insults to promote non obesigenic liver injuries and HCC, we considered alternative strains of mice.

Attempting to recapitulate the human condition without the need for additional liver insult, we used dietary manipulation with the western ‘ALIOS’ diet^18^—previously shown to induce NAFLD and small liver lesions in 60% of C57BL/6 mice at 1 year^14^—but in C3H/He mice. C3H/He mice do develop obesity and hepatomas with age^16,17^. ALIOS fed C3H/He mice developed exaggerated obesity and an elevation in FBG, reflective of insulin resistance. This was associated with human reminiscent NAFLD—including a number of the ancillary features not previously assessed in association with HCC risk. In this model, 96% of ALIOS fed mice developed macroscopic tumours characterised as HCC at 48 weeks of age. Liver weight was markedly higher in ALIOS fed mice, contributed to by more hepatic fat, but also increased hepatocyte proliferation. Each of those features (FBG, liver weight, steatosis and Ki67 proliferation) were significantly associated with tumour development. Notably, the ALIOS diet induced hepatocyte proliferation as early as 24 weeks in the mice, when lesser levels of steatosis were present and NASH was absent. The later acquisition of inflammation, DNA damage and fibrosis were also associated with tumour development. The transcriptional landscape of liver tumours in C3H/He mice resembled a proliferative human HCC-subclass.

Bucillamine reduced DNA damage and fibrosis but had little impact on HCC development. Accepting that there may be uncharacterised mechanistic attributes of bucillamine, it also had little impact on some of the other features associated with ALIOS diet induced HCC development—namely FBG, liver weight, steatosis and inflammation. While DNA damage is mandatory in the development of cancer, these data in combination suggest that beyond DNA damage—regardless of whether there is a little or a lot and whether or not there is fibrosis—the proliferative environment may be key. In the presence of cirrhosis, proliferation occurs as a compensatory regenerative response to progressive hepatocyte injury^5^. Here we have demonstrated the presence of proliferation—even with low levels of DNA/cellular damage and fibrosis—again associated with cancer risk. This is reminiscent of human NAFLD-HCC arising in elderly patients with the metabolic syndrome, in the absence of significant injury and cirrhosis. The cause of the proliferative environment is pertinent and while association studies have their limitations, even at 24 weeks, the ALIOS diet induced elevations in Ki67+ hepatocytes, correlating closely with liver weight (Spearman correlation 0.663, p = 0.008, n = 16) and FBG (0.533, p = 0.033), rather than body weight. At the level of histology, mild to moderate steatosis was present at 24 weeks, without evidence of lobular inflammation or NASH or elevated macrophages on light microscopy. Lipid metabolic pathways are altered when hepatocytes switch to proliferation^33^, although the stimulus, switching hepatocytes to proliferation in the context of steatosis, is not well characterised.

Of note, HCC did not develop in the DINAH model until at the earliest 36 weeks, after the development of additional features including inflammation. In particular, we have highlighted the potential role of lipogranulomas—well recognised as ancillary features in NAFLD—as promoters of an HCC permissive environment. Macrophages are the cardinal immune cell type associated with lipogranulomas, with an elevation in CD68+ macrophages evident from 36 weeks in ALIOS fed mice. In addition—led by our transcriptome analysis of non-tumour fatty liver in C3H/He mice, also highlighting a role for macrophages, we explored a specific candidate, namely CD44, with a suggested role as an upstream regulator of T cell homing. We confirmed that CD44+ was acquired in the livers of mice with HCC, that it was predominantly expressed in macrophages, and that its expression was closely associated with expansion of CD4 T cells which characterise a cancer permissive environment^34–36^. In human NAFLD, CD44 expression in immune cells has been reported to play a key role in NASH^37^ and here we have shown that CD44+ macrophages are acquired in NAFLD patients developing HCC. Furthermore, the elevation in CD44+ macrophages in these patients was associated with the presence of T2DM, which the more modest elevations in CD68 and CD163 positive cells were not. In the contexts of cirrhosis and viral hepatitis, cross talk between macrophages and T cells in the creation of an immunosuppressive environment in which cancers develop and progress, is increasingly recognised^34,35,38^. The DINAH study is an observational one, but highlights these features as also being key in the development of NAFLD-HCC, even in the absence of cirrhosis, where T2DM associated CD44+ macrophage phenotype is acquired. The metabolic and inflammatory relationships between macrophages and their states of activation have been recently reviewed^39^, with the field and identification of disease specific subtypes rapidly advancing^27–29^. Although we did not identify the recently reported NAFLD associated subtypes of CD9 or Trem2+ macrophages as elevated in ALIOS fed mice livers, CD9 was present at transcript and protein levels in both groups at 48 weeks and may have been acquired with age. Trem2 was elevated, but at low levels in whole liver and more sensitive methods may have detected it. However, CD9 and Trem2 have been identified as associated with progressive fibrosis in NAFLD, which is not the most striking phenotype of ALIOS fed C3H/He mice. Going forward, single cell studies in models focused on non-cirrhotic HCC may identify and characterise subtypes in addition to the CD44+ one reported here.

Our aim in conducting this comprehensive observational study was to gain further insights in NAFLD-HCC pathogenesis. In conclusion, the DINAH model recapitulates many of the pathological and histological features of human NASH and HCC. We highlight the importance of elevations in blood glucose and insulin resistance, a proliferative environment and elevated liver weight, alongside a CD44+ macrophage phenotype in fatty livers in which HCC develop. Further evaluation of the metabolic regulation of macrophage phenotype in NAFLD may shed light on preventive or therapeutic strategies for HCC.

## Methods

### Mouse studies

Experiments were approved by the Newcastle Animal Welfare and Ethical Review Board and performed with a UK Home Office licence, according to UK Home Office guidelines. Our study was compliant with ARRIVE guidelines, as summarised in Supplementary Table 5. In a pilot study, 8 week old male C3H/He mice (12 per group) were fed an American lifestyle diet (ALIOS: 45% fat calories, high trans-fat with high fructose drinking water) or a control diet (15% fat calories, low trans-fat without sugar) ad libitum. The diets (ALIOS: Teklad TD.110201; Control: Teklad TD.110196) were distributed from Harlan Laboratories (Madison, Wisconsin, USA). C3H/He mice were C3H/HeNHsd (Harlan, UK) or (C3H/HeNCrl) (Charles River, UK), not C3H/HeJ. C3H/HeJ mice, not the ones we used, are known to be hypo-responsive to lipopolysaccharide consequent to a point mutation in the TLR4 gene^40^. A glucose tolerance test (GTT) was performed in fasted mice at 48 weeks, following which the mice were humanely killed. This dietary induced NASH and HCC (DINAH) model was taken forward in a comprehensive follow-up study, but killing was at 12, 24, 36 (n = 8 per group) and 48 weeks of age (n = 24 per group). In a parallel intervention study, the diets were supplemented with bucillamine 20mg/kg/day from 24 weeks of age until killing at 48 weeks of age. A more detailed overview of the study groups and justification of numbers is provided in Supplementary methods and in Supplementary Table 1. DINAH pilot and comprehensive studies were combined for presentation and statistical analyses.

### Human studies

Informed consent was obtained from patients to use their surplus tissues. Prospectively recruited adult patients were over 18 years of age. The use of their tissues surplus to diagnostic requirements for research purposes was approved by the Newcastle and North Tyneside Regional ethics committee, the Newcastle Academic Health Partners Bioresource (NAHPB) and the Newcastle upon Tyne NHS Foundation Trust Research and Development (R&D) department, in accordance with Health Research Authority guidelines. (References 10/H0906/41; NAHPB Project 48; REC 12/NE/0395; R&D 6579; Human Tissue Act license 12534). The study cohort included 42 NAFLD patients with a median age of 67 years, of whom 27/42 had developed HCC.

### Supplementary methods

Patients, histology and immunohistochemistry studies, Statistical Analyses, Quantification of gene expression, RNA Sequencing and analyses are described in Supplementary Methods. The mouse model RNA sequencing data has been deposited in NCBI’s Gene Expression Omnibus^41^ and is accessible through GEO Series accession number GSE137407 (https://www.ncbi.nlm.nih.gov/geo/query/acc.cgi?acc=GSE137407).

## Supplementary Information

Supplementary Information.
