# Supplementary material for: Key features of the environment promoting liver cancer in the absence of cirrhosis
Source: Sci Rep. 2021 Aug 18;11:16727. doi: 10.1038/s41598-021-96076-2 (PMC8373870; doi:10.1038/s41598-021-96076-2)
Supplement: Supplementary file 1 — Supplementary Information. [file 41598_2021_96076_MOESM1_ESM.pdf]

Key features of the environment promoting liver cancer in the absence of cirrhosis

List of authors

Marco Youssef William Zaki; Ahmed Khairallah Mahdi; Gillian Lucinda Patman; Anna Whitehead; João Pais Maurício; Misti Vanette McCain; Despina Televantou; Sameh Abou-Beih; Eric Ramon-Gil; Robyn Watson; Charlotte Cox; Jack Leslie; Caroline Wilson; Olivier Govaere; John Lunec; Derek Austin Mann; Sirintra Nakjang; Fiona Oakley; Ruchi Shukla; Quentin Mark Anstee; Dina Tiniakos; Helen Louise Reeves.

Supplementary Information

Supplementary Methods, pages 2-7

Supplementary Figures 1-5, pages 8-12

Supplementary Methods Tables, pages 13-16

Supplementary Results Tables, pages 17-20

Supplementary References, page 21

Methods

Animal procedure

The distribution of mice in different dietary and interventional groups is summarised and justified in **Supplementary Table 1**. In the follow-up comprehensive study, mice were housed 4 per cage to reduce fighting injuries. A simple procedure to remove preputial glands was routinely performed at 28 weeks, without complications. Humane killing was at 4 time-points, namely 12 weeks (1 month on diet; n=8 per group), 24 weeks (4 months on diet; n=8 per group); 36 weeks (n=8 per group) and 48 weeks (n=24 per group). An intervention study was performed in parallel, with 12 mice in each dietary group receiving bucillamine (Santen Pharmaceutical, UK) 20mg/kg/day (added to the diet, with dose estimated based on average daily consumption) from 24 weeks of age until humane killing at 48 weeks. Mice were supplied with the stipulation 'no runts', in batches every two to three months over one year. Cage of 4 mice were randomly allocated to different groups from each batch. Blood samples collected before diet initiation and culling had lipid and liver enzymes tested at MRC Harwell; Oxfordshire, UK.

Upon culling, body/liver weight and the macroscopic number and size (using callipers) of tumours were recorded, with technicians blinded to group allocation. Aliquots of liver tissues were snap frozen in liquid nitrogen or placed in RNA later at 4 degrees for 24 hours, prior to storing at -80 degrees. The remainder of the liver tissues were fixed in formalin. Additional analyses are described in the methods and text. All assessments of phenotypic characteristics (e.g. histopathology, immunohistochemistry) were performed with researchers blinded to groups allocation.

Patients

The study cohort included 42 NAFLD patients with a median age of 67 years, of whom 27/42 had developed HCC. Demographic data included age and sex, with body mass index (BMI) and presence of type 2 diabetes (T2DM) recorded. Each of the controls had a documented history of NAFLD, with an otherwise negative liver screen (HBV and HCV serology, serum ferritin, autoantibody screen, alpha-1-antitrypsin level). Patients classed as having NAFLD-HCC were men or women with evidence of a fatty liver on biopsy or imaging or having the presence of at least T2DM or BMI >30, with an otherwise negative liver screen, drinking <21 or 14 units of alcohol per week respectively for at least 5 years prior to their first presentation with liver disease. The diagnosis of HCC was established through non-invasive assessment or histologically according to EASL clinical practice guidelines [1]. Cirrhosis was present in 11/42 (26.2%) of patients. The median body mass index (BMI) of the patients was 31.35 and 23/42 (57.5%) had T2DM.

Tissue studies

Formalin fixed paraffin embedded (FFPE) tissue from tumour and non-tumour liver was sectioned 5 microns thick for histological assessments. An expert liver pathologist (DT) blinded to the dietary groupings assessed histological features of non-tumour and tumour liver tissue. Haematoxylin and eosin (H&E) and Sirius Red staining of the non-neoplastic liver sections were available. The grade of steatosis (0-3), ballooning (0-2) and lobular inflammation (0-3) was quantified and the NAFLD activity score calculated[2]. Other features of liver injury and inflammation (Mallory-Denk bodies, lipogranulomas (cell-free fat droplet surrounded by more than 3 macrophages),

microvesicular steatosis) were also documented and the stage of fibrosis (pericellular, portal/periportal, bridging) was assessed using a five-tiered scoring system[2]. Sirius red slides were also scanned and digitally assessed with Aperio Imagescope software, using a pixel intensity algorithm, applied to a minimum of 30 pericellular/perisinosoidal areas and expressed as pixel intensity per mm^2. Histological assessment of the tumours was performed using H&E and reticulin staining[3] and defined as hepatocellular adenomas or HCC grade 1 or 2. Parameters considered included the presence and degree of nuclear atypia, the presence of cytoplasmic hyaline eosinophilic inclusions and mitotic figures, and the thickness of hepatocyte plates. Additionally, the presence and extent (% of the tumour area) of steatosis, features of degeneration (Mallory-like bodies, ballooning) and inflammation inside the tumours were also assessed.

RNA extraction and Quantification of gene expression

RNA later solution (Invitrogen, Paisley, UK) stored murine liver samples were thawed on ice and RNA was extracted using an RNeasy Mini Kit (Qiagen) according to the manufacturer's protocol. A DNase treatment step using on-column digestion with an RNase-free DNase kit (Qiagen) was included. The RNA concentration and integrity was determined by measuring the optical density (O.D.) at 260 nm with a Nanodrop ND-1000 micro-spectrophotometer (labtech, International). The eluted RNA samples (30 µl) were stored at -80 ^{0}C. Reverse transcription was according to manufacturers protocols (Cat. No. A3500, Promega).

Expression of tumour markers (glypican-3 (GPC3), neighbour of Punc E11 (NOPE) and alpha fetoprotein (AFP)), two inflammatory cytokines (tumour necrosis factor alpha (TNFα), inducible nitric oxide synthase (iNOS)) and collagen 1a1 (col1a1) were analysed by semi-quantitative real time PCR relative to glyceraldehyde-3-phosphate (GAPDH). Primer information is in **Supplementary Table 2A**.

Prior to qPCR, cDNA was thawed on ice and further diluted with 90µl of nuclease-free deionised water to ensure a final concentration of 5 nanogram per microliter. A master mix was prepared for each set of primers, which consisted of the following components for each 10 µl single reaction: 5 µl Platinum SYBR Green qPCR SuperMix-UDG with ROX (Invitrogen), 0.2 µl (10 µM) of each primer (forward and reverse) and 2.6 µl of nuclease-free H_2O. Two microliters of diluted cDNA (10 ng) was added to each single reaction well, with 8 µl of master mix, to get a total volume of 10µl in each well. The qPCR reaction was performed using the 7900HT Fast Real-Time PCR System (Applied Biosystems, Foster city, CA) with the standard thermal cycle steps (50^{0}C for 2 min, 95^{0}C for 10 min followed by 40 cycles of 95^{0}C for 15 sec and 60^{0}C for 1 min), or an Applied Biosystems QS7 machine with standard conditions. Additional 'Minimum Information for Publication of Quantitative PCR' (MIQE) is in **Supplementary Table 2B**.

Glucose tolerance test (GTT)

Animals were fasted overnight to provide a fasting blood glucose level. The animals were then dosed with 2g/kg glucose by intraperitoneal injection and blood glucose was measured at 30, 60 and 120 minutes.

Blood biochemistry

Venous blood was collected from the tail vein before diet initiation. At killing, blood was collected by cardiac puncture into Eppendorf tubes, with plasma collected after centrifugation at 3000 rpm for 10 minutes at room temperature. Plasma was stored at -80°C until lipid and liver enzyme profiling (at MRC Harwell; Oxfordshire, UK).

Immunohistochemistry (IHC) and Western blotting

FFPE tissue sections were immunostained for Ki67, γ-H2AX, CD44, CD68, CD163, F4/80, CD4, FOXP3, CD9, Trem2. Slides were scanned and assessed digitally with Aperio Imagescope Software. The percentage of Ki67 and γ-H2AX positive hepatocyte nuclei was calculated by applying an Aperio imagescope algorithm. T cells and neutrophil counts were manual counts, using the number of cells in 15 high power fields for each case. The macrophage counts were manual counts, quantifying the average numbers of cells per high power field, using a minimum of 10 fields per case. Antibodies used for IHC and western blotting are listed in **Supplementary Table3**.

Dual Staining for CD44 and CD68

FFPE tissue sections (mouse and human) were de-waxed and rehydrated before being submerged in citrate buffer pH 6.0 in an Antigen Access Unit (A. Menarini diagnostics, Berkshire, UK). Immunofluorescence (IF) was performed as per standard protocols sequentially with anti-CD44 followed by anti-CD68 antibodies. Details of antibodies utilised is given in **Supplementary Table 4**. IF slides were counterstained with DAPI (1:1000 for 30 min) and mounted using Vector TrueVIEW Autofluorescence quenching kit (SP-8400) as per the instructions. Images were captured using a confocal microscope (Leica TCS SPE) at 40X lenses with additional 1.5x zoom and analysed by Fiji ImageJ.

RNA sequencing, statistics and data analysis

10-20 mg of non-tumour and tumour tissues were shipped in RNAlater on dry ice to AROS Applied Technology (Denmark), where RNA was extracted and the quality of each sample checked using a Bioanalyser (Agilent). Only samples with at least 400ng of total RNA and a RNA Integrity Number (RIN) greater than 8.0 were included. RNA sequencing was carried out using Illumina's Stranded mRNA kit for library preparation, with 100 bp Paired End Reads, five samples to be sequenced across one lane. RNA sequence analyses were performed with the Newcastle Bioinformatics Support Unit. In short, the RNA-seq Paired-end reads were mapped to the mouse reference genome (mm10) using STAR, with 2-pass alignment mode. HTSeq was used to count the number of reads that overlap with annotated gene features from Gencode version M6, with options -t exon -m intersection-nonempty. Gene-level differential expression analysis was performed using DESeq2[4]. Similarities between samples were determined by unsupervised clustering using hClust function with ward.D2 method in RStudio. Data visualisation was performed using Heatmapper software[5], while deregulated pathways were analysed using both Ingenuity pathway analysis, IPA (QIAGEN Inc., https://www.qiagenbioinformatics.com/products/ingenuitypathway-analysis) and gene set enrichment analysis (GSEA) software [6, 7]. Statistics values were used as a ranking metrics for analysing gene sets acquired from the Molecular Signatures Database (MSigDB v6.2)[8]. Gene sets with <5 or >2000 genes were excluded.

Non-negative matrix factorization analysis

Non-negative Matrix Factorization (NMF) was used to extract metagenes from gene expression profiles of human liver hepatocellular carcinomas of different etiologies, using RNA-seq data downloaded from The Cancer Genome Atlas Hepatocellular Carcinoma (TCGA-LIHC)[9]. A matrix of gene expression data combining per gene read counts of samples from TCGA was normalized and variance-stabilized transformed using DESeq2[4]. To remove genes that did not vary sufficiently across the dataset we capped expression values at 20 and 10,000 read counts and only included genes between > 10-fold minimum and >1000 maximum read counts. To identify the most robust combination of metagenes and subgroups/clusters in the dataset, consensus bootstrapped NMF clustering was performed on the filtered gene count matrix as previously described[10]. Briefly, we performed NMF and K-means clustering, testing all combination of 2-15 metagenes and clusters with bootstrapped resampling method (n=100) to test for reproducibility. Cluster stability measures (Cohen's kappa, average silhouette scores) were assessed to determine optimal combinations of metagenes and clusters. Samples that were assigned to the same cluster fewer than 90% of replicates were removed from the dataset. All genes in the refined dataset were then column rank normalised and metagenes scores were recalculated. Metagenes derived from the human HCC dataset were then projected onto our own NAFLD and HCC mouse model RNA-seq dataset by the pseudoinverse method as previously described[11, 12]. All analysis and figures were generated using R/Bioconductor and by using a modified version of the NMF scripts provided by Brunet et al.[11]. GSEA[7] was performed on a gene list ranked according to the correlation of the metagene scores with the expression profile.

Statistical analyses

Statistical analyses were with SPSS for windows, version 25 (SPSS Inc. Chicago Illinois, USA), licensed to Newcastle University. Data are show as a mean ± standard error of the mean (SEM), as associations, or as categorical scores. Differences between groups of continuous variables were assessed by t-test or ANOVA for parametric data, or Mann-Whitney and Kruskal Wallis tests for non-parametric data. Associations between groups were assessed by Pearson (parametric data) or Spearman (non-parametric data) tests. Differences between categorical variables were assessed by Pearson Chi Square, or Fishers Exact tests approximated using a Monte Carlo approach where cells within a contingency table of greater than 2x2 contained low numbers (<5). A p-value of <0.05 was considered significant.

Figures

Supplementary Diet Sheet 1 - ALIOS diet TD 110202

Teklad Custom Research Diet Data Sheet

TD.110201 22% HVO Diet (VI)

Formula	g/Kg
Casein	230.0
DL-Methionine	3.4
Sucrose, fine ground	212.9277
Corn Starch	80.0
Maltodextrin	140.0
Vegetable Shortening, hydrogenated (Primex)	220.0
Soybean Oil	10.0
Cellulose	50.0
Mineral Mix, AIN-93G-MX (94046)	46.0
Calcium Phosphate, dibasic	3.3
Niacin	0.063
Calcium Pantothenate	0.0336
Pyridoxine HCl	0.0147
Thiamin HCl	0.0126
Riboflavin	0.0126
Folic Acid	0.0042
Biotin	0.0005
Vitamin B_{12} (0.1% in mannitol)	0.0525
Vitamin E, DL-alpha tocopheryl acetate (500 IU/g)	0.15
Vitamin A Palmitate (500,000 IU/g)	0.0168
Vitamin D_3, cholecalciferol (500,000 IU/g)	0.0042
Vitamin K_1, phylloquinone	0.0016
Choline Bitartrate	3.96
TBHQ, antioxidant	0.046

Footnote

Modification of TD.06303 with vitamins increased for irradiation. Hydrogenated vegetable oil (HVO) is about 30% trans-fat, giving this diet approximately 6.6% trans-fat by weight.

Selected Nutrient Information[1]

	% by weight	% kcal from
Protein	20.4	17.7
Carbohydrate	42.8	37.1
Fat	23.2	45.2
Kcal/g	4.6	

[1] Values are calculated from ingredient analysis or manufacturer data

Teklad Diets are designed & manufactured for research purposes only.

Speak With A Nutritionist

- (800) 483-5523
- askanutritionist@harlan.com

harlan

Harlan Laboratories · PO Box 44220 · Madison, WI 53744-4220
www.harlan.com

Key Features

- Purified Diet
- High Trans Fat
- Suitable for Irradiation

Key Planning Information

- *Products are made fresh to order*
- Store product at 4°C or lower
- Use within 6 months (applicable to most diets)
- Box labeled with product name, manufacturing date, and lot number
- Replace diet at minimum once per week
  More frequent replacement may be advised
- Lead time:
  · 2 weeks non-irradiated
  · 4 weeks irradiated

Product Specific Information

- 1/2" Pellet or Powder (free flowing)
- Minimum order 3 Kg
- Irradiation available upon request

Options (Fees Will Apply)

- Rush order (pending availability)
- Irradiation (see Product Specific Information)
- Vacuum packaging (1 and 2 Kg)

International Inquiry

·Outside U.S.A. or Canada·

- askanutritionist@harlan.com

Place Your Order (U.S.A. & Canada)

· Place Order · Obtain Pricing ·
· Check Order Status ·

- (800) 483-5523
- (608) 277-2066 facsimile
- tekladinfo@harlan.com

Helping you do research better

Harlan, Harlan Laboratories, Helping you do research better, and the Harlan logo are trademarks and trade names of Harlan Laboratories, Inc.
© 2008 Harlan Laboratories, Inc.

Supplementary Diet Sheet 2 - Control TD 110196

Teklad Custom Research Diet Data Sheet

TD.110196 6% Soybean Oil Diet

Formula	g/Kg
Casein	183.0
DL-Methionine	2.7
Sucrose, fine ground	100.0
Corn Starch	431.2751
Maltodextrin	130.0
Soybean Oil	60.0
Cellulose	50.0
Mineral Mix, AIN-93G-MX (94046)	36.8
Calcium Phosphate, dibasic	2.72
Niacin	0.051
Calcium Pantothenate	0.027
Pyridoxine HCl	0.012
Thiamin HCl	0.0105
Riboflavin	0.0105
Folic Acid	0.0033
Biotin	0.0003
Vitamin B_{12} (0.1% in mannitol)	0.042
Vitamin E, DL-alpha tocopheryl acetate (500 IU/g)	0.15
Vitamin A Palmitate (500,000 IU/g)	0.0135
Vitamin D_3, cholecalciferol (500,000 IU/g)	0.0033
Vitamin K_1, phylloquinone	0.0015
Choline Bitartrate	3.17
TBHQ, antioxidant	0.01

Footnote
A control for TD.110201 without trans fat and with reduced sucrose. Protein, minerals and vitamins are adjusted so that they are equivalent to levels in TD.110201 when compared on the basis of kcal density. The exception is vitamin E which is kept the same in both diets (because it was intentionally reduced in TD.06303, which these diets are based on).

Selected Nutrient Information[1]

	% by weight	% kcal from
**Protein**	16.2	17.6
**Carbohydrate**	62.0	67.3
**Fat**	6.2	15.1

Kcal/g 3.7

[1] Values are calculated from ingredient analysis or manufacturer data

Teklad Diets are designed & manufactured for research purposes only.

Speak With A Nutritionist
- **(800) 483-5523**
- **askanutritionist@harlan.com**

Harlan Laboratories · PO Box 44220 · Madison, WI 53744-4220
www.harlan.com

Key Features
- **Purified Diet**
- **Normal Fat Level**
- **No Trans Fat**
- **Control**

Key Planning Information
- *Products are made fresh to order*
- Store product at 4°C or lower
- Use within 6 months (applicable to most diets)
- Box labeled with product name, manufacturing date, and lot number
- Replace diet at minimum once per week
  More frequent replacement may be advised
- Lead time:
  · 2 weeks non-irradiated
  · 4 weeks irradiated

Product Specific Information
- 1/2" Pellet or Powder (free flowing)
- Minimum order 3 Kg
- Irradiation available upon request

Options (Fees Will Apply)
- Rush order (pending availability)
- Irradiation (see Product Specific Information)
- Vacuum packaging (1 and 2 Kg)

International Inquiry
·Outside U.S.A. or Canada·
- **askanutritionist@harlan.com**

Place Your Order (U.S.A. & Canada)
· Place Order · Obtain Pricing ·
· Check Order Status ·
- **(800) 483-5523**
- **(608) 277-2066** *facsimile*
- **tekladinfo@harlan.com**

Helping you do research better

Harlan, Harlan Laboratories, Helping you do research better,
and the Harlan logo are trademarks and trade names of Harlan Laboratories, Inc.
© 2008 Harlan Laboratories, Inc.

Supplementary Figure 1: DINAH model phenotype supplementary

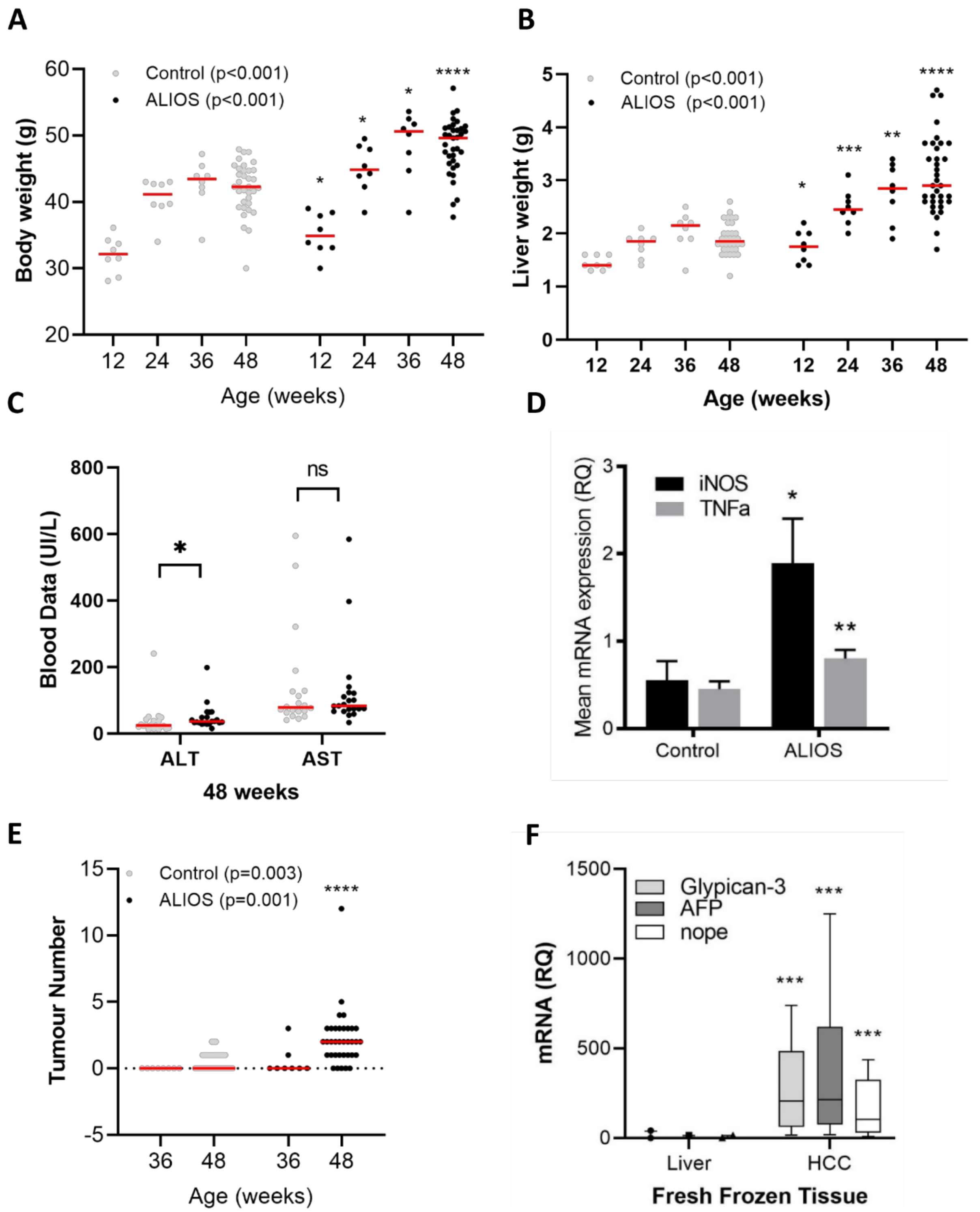

Body and liver weight increased with age in controls, but more so in ALIOS fed mice **(A, B)**. Serum ALT rather than AST was elevated in ALIOS fed mice **(C)**. iNOS and TNFalpha mRNA levels were elevated in ALIOS fed mice **(D)**. Elevations in tumour numbers are summarised in **(E)**. Expression of glypican-3, alfa-fetoprotein and nope tumour markers were elevated in tumour tissues **(F)**. Data are shown as mean±S.E.M, with data analysed by Mann-Whitney or Kruskall Wallis (KW) tests. P values * p<0.05; ** p<0.01; *** p<0.001, **** p<0.0001.

Supplementary Figure 2: Cluster dendogram of the RNASeq hepatic gene expression of the DINAH non-tumour and tumour tissues

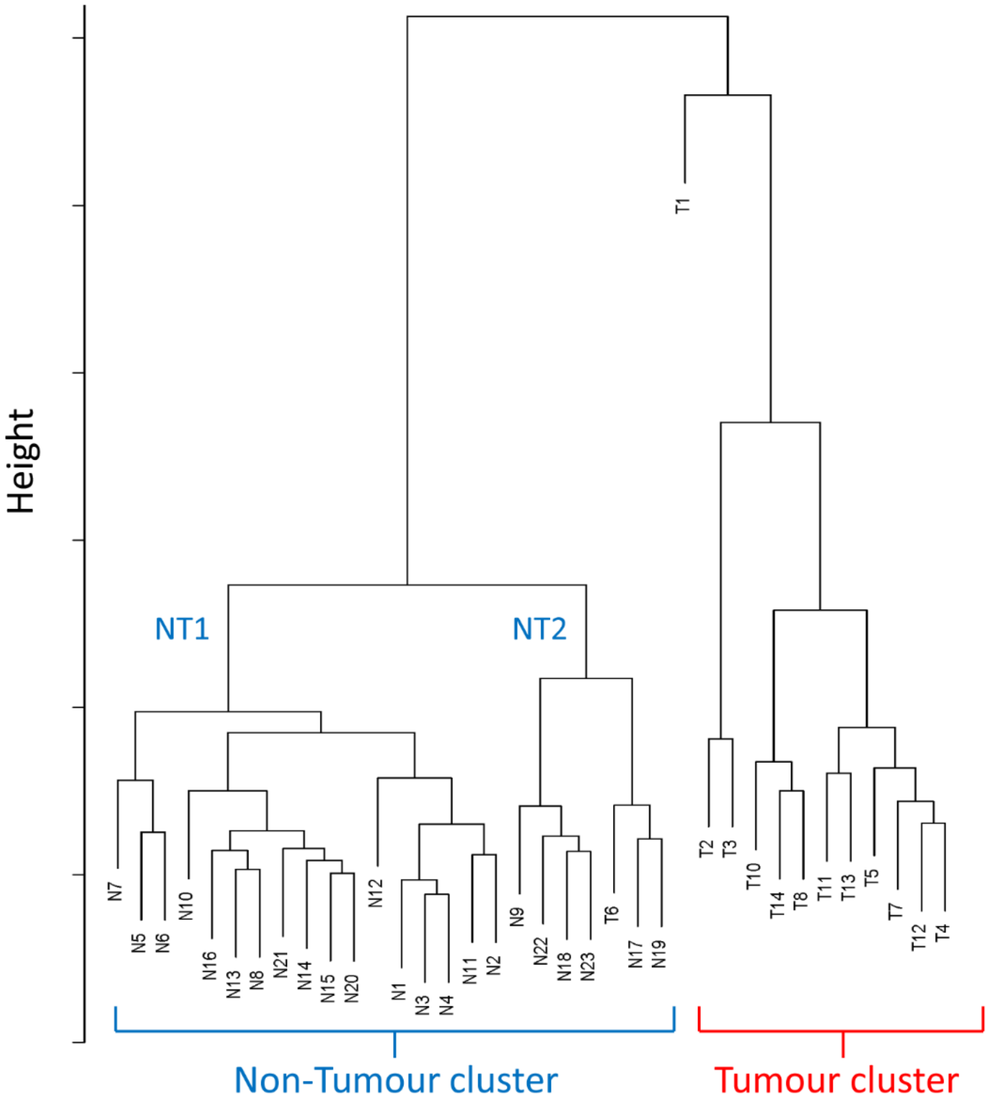

Hierarchical clustering identified two main clusters of the liver transcriptome, largely differentiating the non-tumour (NT) and tumour (T) tissues. In the NT cluster, N1-N12 were from control diet C3H/HeH mice, while N12-N23 were from ALIOS-fed mice. A sub-cluster (NT2) contained more ALIOS fed mice tissues and a single tumour sample (T6) – closer to N17 and N19 in the distance matrix than T2. In the tumour cluster, T1-T14 were tumour tissues from ALIOS-fed mice, except for T7, which was from a control-fed mouse. The outlier T1, was not included in the DINAH mouse tumour transcriptome analyses with human metagenes (Figure 1).

Supplementary Figure 3: Bucillamine – additional information

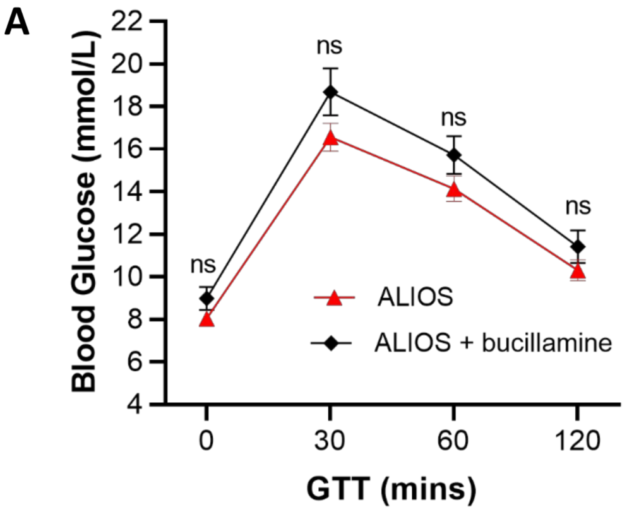

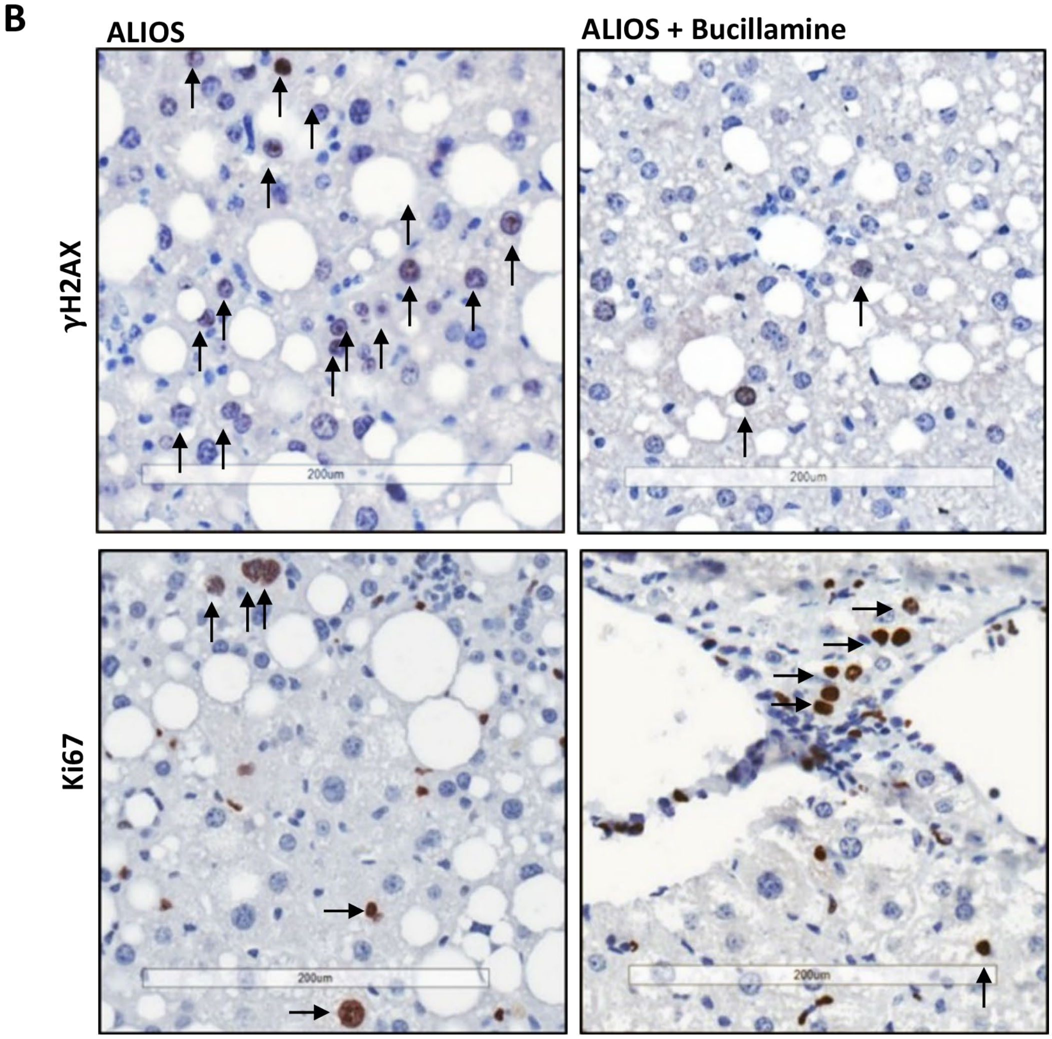

Bucillamine had no significant impact on glucose tolerance test, with data in 48 weeks ALIOS fed mice shown **(A).** Immunohistochemistry detecting γH2AX or Ki67 positive nuclei, annotated with black arrows, is shown in **(B),** in 48 weeks mice fed either ALIOS diet or ALIOS diet supplemented with bucillamine. Bucillamine diminished γH2AX, but had little impact on Ki67 positive nuclei.

Supplementary Figure 4: Phenotype and histological characterisation of the RNA-Seq of non-tumour NAFLD liver G1 versus G2 Clusters

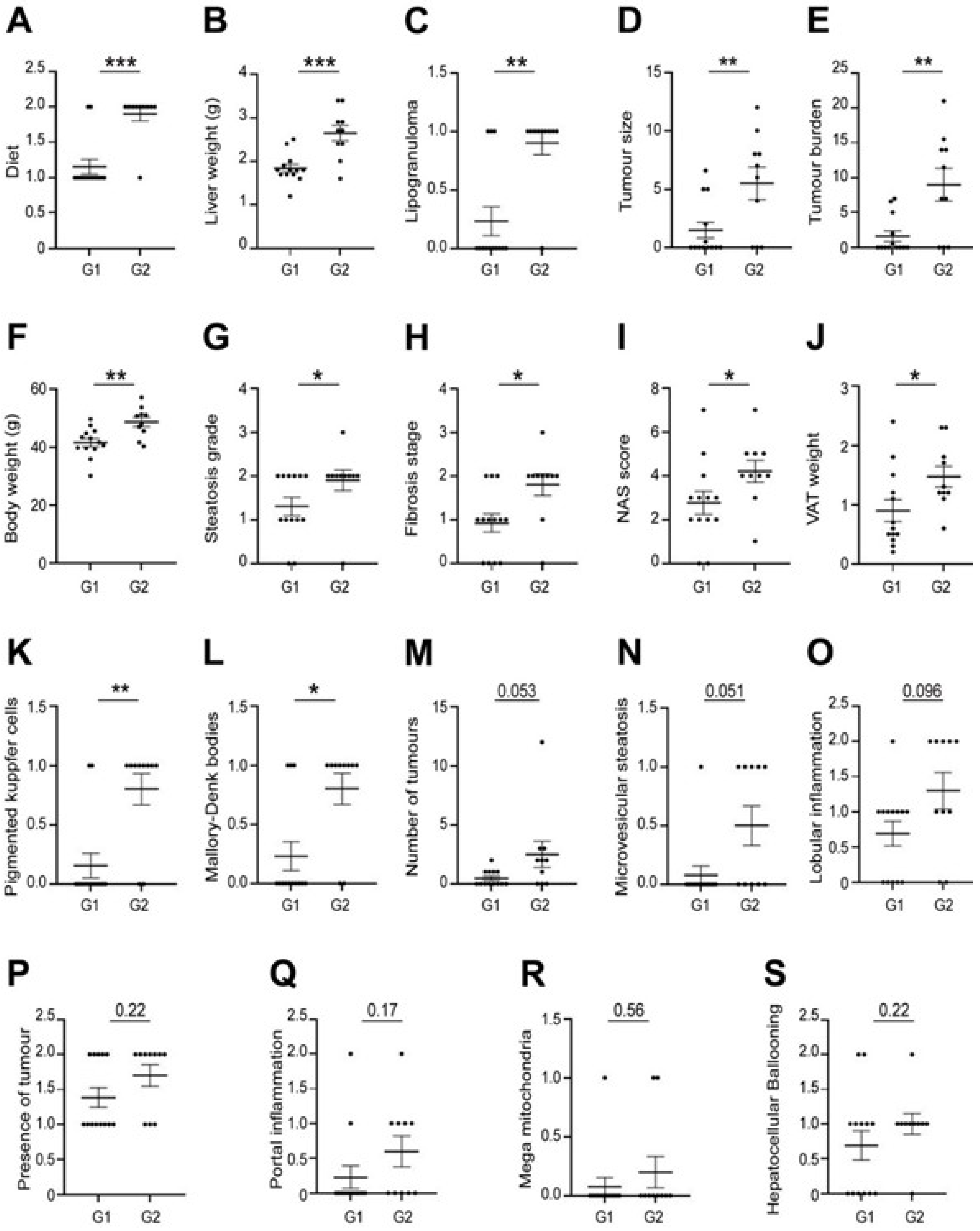

The figure shows the differences between G1 and G2 mice, for dietary intake **(A)**, Liver weight **(B)**, Lipogranuloma **(C)**, tumour size **(D)**, tumour burden **(E)**, body weight **(F)**, steatosis grade **(G)**, stage of fibrosis **(H)**, NAS score **(I)**, VAT weight **(J)**, pigmented Kupffer cells **(K)**, Mallory-Denk bodies **(L)**, tumour number **(M)**, Microvesicular steatosis **(N)**, lobular inflammation **(O)**, presence of tumour **(P)**, portal inflammation **(Q)**, Mega mitochondria **(R)** and hepatocellular ballooning **(S)**. Data are presented as mean± S.E.M, * $p<0.05$; ** $p<0.01$, *** $p=0.001$ (Mann Whitney Tests).

Supplementary Figure 5: Non-tumour fatty liver expression of CD44

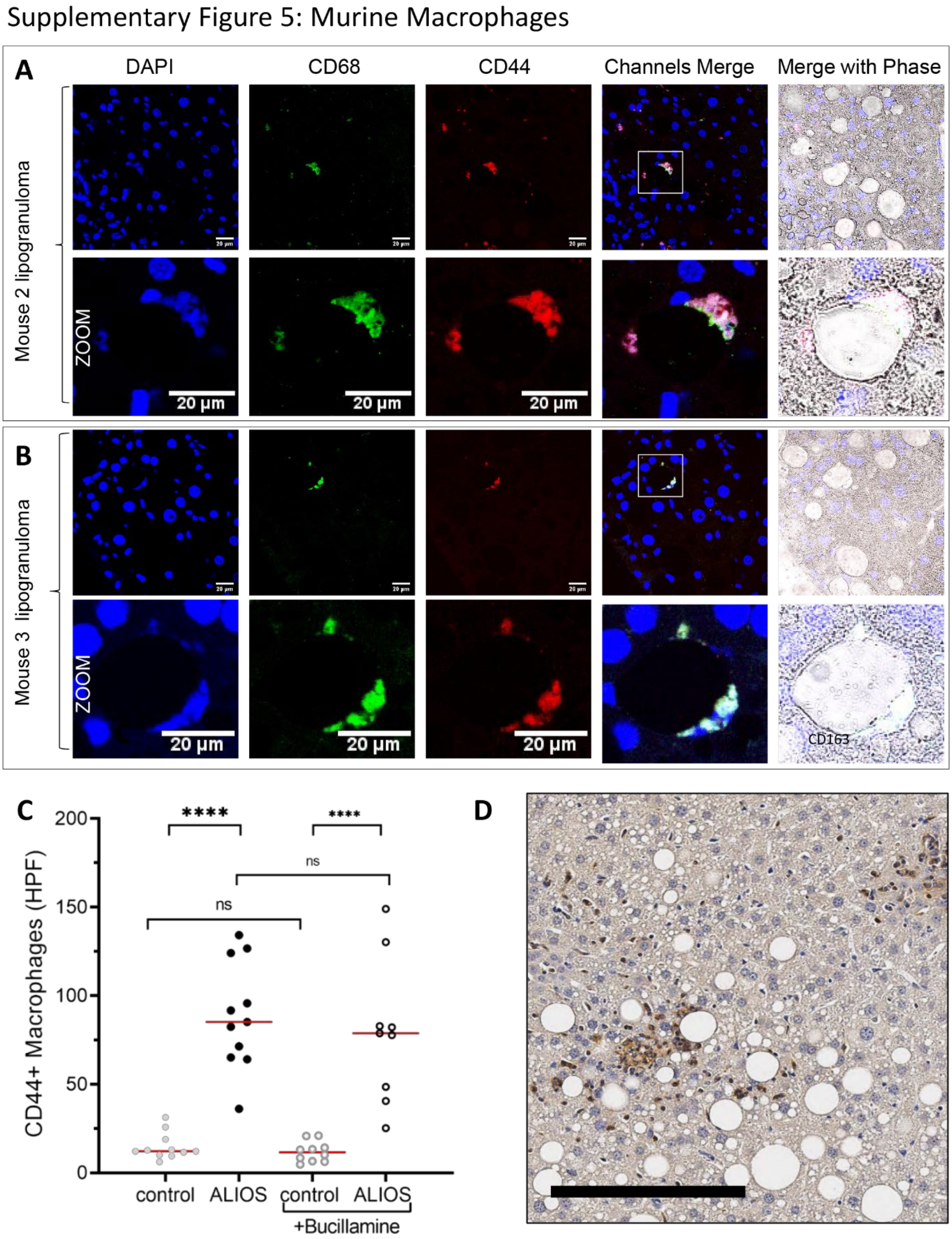

CD44 was co-expressed in macrophages in lipogranulomas, as demonstrated in two additional C3H/He ALIOS fed mice **(A,B).** ALIOS diet induced elevations in CD44+ macrophages at 48 weeks were not altered by bucillamine supplementaion. **(C,D)**.

Tables

Supplementary Methods Table 1 – An overview of mice groups and numbers

**C3H/Heh mice study groups (Total 144)**								
	**Control Diet**				**ALIOS diet**			
Weeks	12	24	36	48	12	24	36	48
Pilot Study				12 (11)				12 (11)
Main Study	8 (8)	8 (8)	8 (8)	24 (23)	8 (8)	8 (8)	8 (8)	24 (24)
Main +Bucillamine				12 (11)				12 (11)
Pilot + Main				36 (34)				36 (35)

In the pilot study, 12 mice per group were included, with survivors killed at 48 weeks. The numbers of mice reaching the planned cull are shown in brackets. In the more comprehensive follow-up study, 8 mice per group were included for planned cull time points 12-36 weeks on either control or ALIOS diets. For the 48 weeks time point in the main study, where we aimed to consider phenotypic and histological features associated with tumour development, significant heterogeneity was expected, based on the pilot study. Estimating 45-50% of the control group would develop age associated phenotypic changes (including tumours), with that proportion increased to 85-90% in the ALIOS group, in order to detect significant differences with 80% power and 5% error, including a 10-15% attrition rate, we included 24 mice per group. The final row shows the combined numbers of mice in pilot and main studies included in the phenotypic studies.

Supplementary Methods Table 2A - PCR Primer information

**Gene Symbol**	**Accession no.**	**Primer direction, sequence (5'-3'), amplicon length (bp)**		
**Gpc3**	NM_016697.3	Forward	ATTGGAAGCTCTGGTGACG	83
		Reverse	TCCACATCCAGATCATAGGC	
**Igdcc4 (nope)**	XM_036155152.1	Forward	GCCATGGATGCCTTAAGTTC	102
		Reverse	AGCCAGGTAAACGAGTGAGC	
**Afp**	NM_007423.4	Forward	CCAGGAAGTCTGTTTCACAGAAG	130
		Reverse	CAAAAGGCTCACACCAAAGAG	
**Tnfα**	NM_013693.3	Forward	ATGAGAAGTTCCCAAATGGC	125
		Reverse	CTCCACTTGGTGGTTTGCTA	
**Nos2 (iNos)**	NM_001313922.1	Forward	CTTTGCCACGGACGAGAC	66
		Reverse	TCATTGTACTCTGAGGGCTGAC	
**Col1a1**	NM_007742.4	Forward	TTCACCTACAGCACCCTTG	66
		Reverse	GATGACTGTCTTGCCCCAA	
**Gapdh**	NM_001289726.1	Forward	GGTGCTGAGTATGTCGTGGAGTCTA	100
		Reverse	GGGCGGAGATGATGACCCTTT	

Supplementary Methods Table 2B – Additional MIQE PCR information

**Definition of experimental and control groups, numbers within groups and descriptions** are as described in manuscript and figure legends	
Processing procedure	Detailed in supplementary methods
Samples freezing	RNA later 24 hours 4 degrees then -80
Samples storage and duration	Stored in -80. RNA extraction, PCR was within 1-2 year Recent PCR on cDNA stored for 2 years.
**Nucleic acid extraction**	
Procedure described in supplementary methods	
RNA integrity	Bioanalyser when additionally used for RNAseq
Inhibition testing	The standard curve
**Reverse transcription** is described in supplementary methods	
**qPCR target info**	
Gene symbol; accession number ; Amplicon length in Table 2A	
specificity screen	NCBI primer blast
Location exon intron	Junction spanning primers
Splice variants targeted - none	
**qPCR oligonucleotides**	
Primer sequences	Table 2A
location and identity of any modifications - none	
**qPCR protocol** is as described in methods	
**qPCR validation**	
Specificity,	Melt curve analysis performed for each sample
Calibration curve	completed and calculated the efficiency of the primer pair
r2 of calibration curve	Calculated for each primer pair
linear dynamic range	Curves were calculated from 3-4 dilutions
Cq variation at LOD	NA
Evidence for LOD	Instead of LOD, we utilised primer standard curves to make sure that the samples Cq fall in the linear range
**Data analysis**	
qPCR analysis	QuantStudio
Method of Cq	Determined as above
outliers	variation in Cq - discarded from analysis.
results for NTCs	The signal of the amplification plot was mostly undetermined or very late (Cq>34).
Justification reference genes	GAPDH used as cQ values for this was very similar between all samples analysed within an assay.
normalisation method	Described in methods and/or figure legends
technical replicates	Three were run per sample on each qPCR plate
repeatability	Multiple biological replicate samples, which were repeatable. In addition RNAseq data confirms qPCR data.
statistical methods	See methods and fig. Legends for details
software	Microsoft Excel

Supplementary Methods Table 3 – Antibodies for immunohistochemistry or western blot

Staining	Catalogue number	Company	Clonality/ species	Antigen retrieval	Experiment
Ki67	ab16667 (SP6)	Abcam	Rabbit mAb	EDTA, 1mM	IHC (1:1000)
gamma-H2AX	9718S	Cell signalling	Rabbit mAb	EDTA, 1mM	IHC(1:100)
Pan CD44	ab157107	Abcam	Rabbit pAb	Citrate buffer (pH6)	IHC (1:1000)
CD68	OABB00472	Aviva systems biology	Rabbit pAb	Citrate buffer (pH6)	IHC (1:200)
CD163	760-4437	Roche	Mouse mAb	CC1 (Roche)	IHC (Roche)
F4/80	Ab6640	Abcam	Rat mAb	20ug/ml Proteinase-K	IHC (1:100)
CD4	25229S	Cell signalling	Rabbit mAb	Citrate buffer (pH6)	IHC (1:100)
FOXP3	14-5773-82	Ebioscien ces	Rat mAb	EDTA, 1mM	IHC (1:100)
Phospho-p44/42 MAPK (ERK1/2)(Thr2 02/Tyr204)	9101	Cell signalling	Rabbit pAb		WB (1:1000)
p44/42 MAPK (ERK1/2)	9102	Cell signalling	Rabbit pAb		WB (1:1000)
GAPDH	ab37168	Abcam	Rabbit pAb		WB (1:2000)
TREM2	MAB17291	R&D Systems	Rat mAb	HIER Universal (Abcam)	IHC (1:100)
CD9	AB223052	Abcam	Rabbit pAb	CC1 (Roche)	IHC (1:250)

Supplementary Methods Table 4 – Antibodies for Dual labelling macrophages

Target Tissue	Target Protein	Antibody	Company and Species	Dilution for IF	Secondary Antibody for IF
Mouse and Human	Pan CD44	ab157107	Abcam Rabbit pAb	1:10	AF 546 Anti rabbit 1:200 Invitrogen™/Thermo Fisher Scientific
Mouse	CD68	OABB00472	Aviva systems biology Rabbit pAb	1:200	AF 488 anti-rabbit 1:200 Invitrogen™/Thermo Fisher Scientific
Human	CD68	MA5-13324	ThermoFisher Mouse mAb	1:50	AF 488 anti-mouse 1:200 Invitrogen™/Thermo Fisher Scientific

Supplementary Methods Table 5

The ARRIVE guidelines 2.0: author checklist

The ARRIVE Essential 10

These items are the basic minimum to include in a manuscript. Without this information, readers and reviewers cannot assess the reliability of the findings.

Item		Recommendation	Section/line number, or reason for not reporting
**Study design**	1	For each experiment, provide brief details of study design including:<br>a. The groups being compared, including control groups. If no control group has been used, the rationale should be stated.<br>b. The experimental unit (e.g. a single animal, litter, or cage of animals).	a)Main text, methods section, plus Supplementary methods page 2, Supplementary Table 1 (page 13)<br>b) Single unit
**Sample size**	2	a. Specify the exact number of experimental units allocated to each group, and the total number in each experiment. Also indicate the total number of animals used.<br>b. Explain how the sample size was decided. Provide details of any *a priori* sample size calculation, if done.	a) Main text, methods section, plus Supplementary methods page 2, Supplementary Table 1 (page 13)<br>b) Supplementary methods, page 13
**Inclusion and exclusion criteria**	3	a. Describe any criteria used for including and excluding animals (or experimental units) during the experiment, and data points during the analysis. Specify if these criteria were established *a priori*. If no criteria were set, state this explicitly.<br>b. For each experimental group, report any animals, experimental units or data points not included in the analysis and explain why. If there were no exclusions, state so.<br>c. For each analysis, report the exact value of *n* in each experimental group.	When ordering animals, specification 'no runts' was made. No other criteria.<br>All animals were used in the generation of data.<br>Supplementary Table 1, page 13
**Randomisation**	4	a. State whether randomisation was used to allocate experimental units to control and treatment groups. If done, provide the method used to generate the randomisation sequence.<br>b. Describe the strategy used to minimise potential confounders such as the order of treatments and measurements, or animal/cage location. If confounders were not controlled, state this explicitly.	a) Cages of 4 animals were allocated to specific dietary groups. The dietary groups comprised 8-24 animals.<br>a) On delivery of batches of animals, a cage of 4 was allocated to distinct dietary groups i.e. shared between groups, not one entire group from one batch delivery.
**Blinding**	5	Describe who was aware of the group allocation at the different stages of the experiment (during the allocation, the conduct of the experiment, the outcome assessment, and the data analysis).	The animal technicians responsible for feeding and handling the animals. Researchers collecting outcome data were not aware.
**Outcome measures**	6	a. Clearly define all outcome measures assessed (e.g. cell death, molecular markers, or behavioural changes).<br>b. For hypothesis-testing studies, specify the primary outcome measure, i.e. the outcome measure that was used to determine the sample size.	Body weight, liver weight, blood anaylses, liver histopathology, RNA sequencing data. In main text, these are Figures 1-5 and Tables 1-2.<br>Supplementary methods, page 13
**Statistical methods**	7	a. Provide details of the statistical methods used for each analysis, including software used.<br>b. Describe any methods used to assess whether the data met the assumptions of the statistical approach, and what was done if the assumptions were not met.	Supplemeentary methods pages 4-5<br>none
**Experimental animals**	8	a. Provide species-appropriate details of the animals used, including species, strain and substrain, sex, age or developmental stage, and, if relevant, weight.<br>b. Provide further relevant information on the provenance of animals, health/immune status, genetic modification status, genotype, and any previous procedures.	Supplementary methods page 2, Supplementary Table 1 (page 13)<br>As above, plus results section one, lines 2-4
**Experimental procedures**	9	For each experimental group, including controls, describe the procedures in enough detail to allow others to replicate them, including:<br>a. What was done, how it was done and what was used.<br>b. When and how often.<br>c. Where (including detail of any acclimatisation periods).<br>d. Why (provide rationale for procedures).	a) weight, glucose tolerance test (GTT), blood sampling, culling & tissues collection.<br>b) Weight weekly. GTT and blood sampling age 8-9 weeks and age 47-48 weeks. Culling and tissues collection at time periods indicated (12, 24, 36, 48 weeks)<br>c) Home Office licensed animal house<br>d)To study features associated with development of disease with age
**Results**	10	For each experiment conducted, including independent replications, report:<br>a. Summary/descriptive statistics for each experimental group, with a measure of variability where applicable (e.g. mean and SD, or median and range).<br>b. If applicable, the effect size with a confidence interval.	a) A pilot study determined the numbers of mice used in the main study groups.<br>b) Statistics and confidance intervals are reported in Figures and Tables.

Supplementary Results Table 1. Histological characterisation of the DINAH model time course

Classical features for diagnosis and severity assessment in NAFLD						
**Diet**	**Control**	**ALIOS**	**p value**	**Control**	**ALIOS**	**p value**
**Steatosis grade (0/1/2/3)**				**Hepatocellular ballooning (0/1/2)**		
**12 weeks**	4/4/0/0	0/5/3/0	0.020	6/2/0	6/2/0	ns
**24 weeks**	0/1/7/0	0/2/5/1	ns	2/6/0	2/3/3	ns
**36 weeks**	2/2/4/0	0/0/6/2	0.030	3/4/1	5/3/0	ns
**48 weeks**	4/10/9/0	0/1/19/4	<0.001	8/11/4	1/21/2	ns
**Lobular Inflammation score (0/1/2)**				**NAFLD activity (NAS) score**		
**12 weeks**	4/3/1	5/3/0	ns	1.4 ± 0.4	2.1 ± 0.4	ns
**24 weeks**	7/1/0	5/3/0	ns	2.8 ± 0.3	3.5 ± 0.4	ns
**36 weeks**	6/1/1	2/3/3	ns	2.4 ± 0.4	3.6 ± 0.4	0.07
**48 weeks**	12/11/0	1/17/6	<0.001	2.5 ± 0.3	4.5 ± 0.2	<0.001
**Portal Inflammation and Fibrosis scores**				**Ancillary Features of NAFLD**		
**Portal Inflammation score (0/1/2)**				**Lipogranuloma (0/1)**		
**12 weeks**	1/6/1	2/2/4	ns	8/0	8/0	ns
**24 weeks**	3/5/0	2/4/2	ns	8/0	8/0	ns
**36 weeks**	0/6/2	0/1/7	0.041	8/0	8/0	ns
**48 weeks**	21/2/0	18/4/2	ns	22/1	3/21	<0.001
**Peri-sinusoidal fibrosis score (0/1)**				**Microvesicular steatosis (0/1)**		
**12 weeks**				8/0	8/0	ns
**24 weeks**	0/8	0/8	ns	8/0	7/1	ns
**36 weeks**				8/0	8/0	ns
**48 weeks**	7/16	0/24	0.004	23/0	16/8	0.004
**Peri-portal fibrosis score (0/1/2)**				**Pigmented Kupffer cells (0/1)**		
**12 weeks**				8/0	8/0	ns
**24 weeks**	3/5	0/8	ns	8/0	8/0	ns
**36 weeks**				8/0	7/1	ns
**48 weeks**	17/6/0	12/11/1	ns	16/7	9/15	0.045
**Fibrosis stage (0/1/2/3)**				**Mallory Denk bodies (0/1)**		
**12 weeks**				8/0	8/0	ns
**24 weeks**	0/6/2/0	0/1/7/0	0.041	8/0	8/0	ns
**36 weeks**				8/0	7/1	ns
**48 weeks**	7/10/6/0	0/12/11/1	0.009	18/5	10/14	0.019
				**Megamitochondria (0/1)**		
				8/0	8/0	ns
				8/0	8/0	ns
				8/0	8/0	ns
				20/3	14/10	0.0509

Supplementary Results Table 2. The DINAH Bucillamine intervention study

	Control diet			ALIOS diet		
	**Control**	**+ Bucill-amine**	**p**	**ALIOS**	**+ Bucill -amine**	p
**number**	23	11		24	11	
**Phenotype features**						
Body weight (g)	41.3±0.83	41.9±1.52	ns	47.25±0.92	45.1±1.21	ns
Liver weight (g)	1.85±0.06	1.98±0.12	ns	2.84±0.13	2.66±0.15	ns
Liver/BW ratio	0.04±0.00	0.05±0.00	ns	0.06±0.00	0.06±0.00	ns
VAT (g)	0.87±0.10	0.88±0.13	ns	2.00±0.13	1.85±0.17	ns
FBG (mmol/l)	6.72±0.19	7.47±0.47	ns	8.05±0.32	8.18±0.21	ns
Total Cholesterol	5.51±0.19	5.67±0.33	ns	5.65±0.27	5.75±0.39	ns
LDL (mmol/l)	0.97±0.05	1.38±0.17	ns	1.11±0.08	1.21±0.15	ns
HDL (mmol/l)	3.52±0.18	3.39±0.37	ns	3.47±0.20	3.48±0.42	ns
ALT (IU/L)	37.9±11.01	64.4±15.88	0.028	49.1±8.39	58.1±5.26	ns
**Histology Features**						
Steatosis (0/1/2/3)	4/10/9/0	7/4/0/0	0.007	0/1/19/4	1/2/6/2	ns
MV steatosis (0/1)	23/0	11/0	NA	16/18	9/2	ns
Ballooning (0/1/2)	8/11/4	11/0/0	0.001	1/21/2	11/0/0	<0.001
MDB (0/1)	17/5	11/0	ns	10/14	11/0	0.001
Megamitochondria (0/1)	19/3	11/0	ns	14/10	9/2	ns
Lipogranulomas (0/1)	21/1	8/3	ns	3/21	1/10	ns
LI (0/1/2/3)	12/11/0/0	0/11/0/0	0.003	1/17/6/0	1/5/4/1	ns
PI (0/1/2)	21/2/0	10/1/0	0 ns	18/4/2	7/2/2	ns
PK cells (0/1)	15/7	11/0	0.035	9/15	8/3	0.053
Apoptotic cells (0/1)	19/3	10/1	ns	18/6	9/2	ns
PS fibrosis (0/1)	7/16	7/3	0.035	0/24	6/4	<0.001
PP fibrosis (0/1/2)	17/6/0	10/0/0	0.074	12/11/1	7/3/0	ns
Fibrosis stage (0/1/2/3)	7/10/6/0	7/3/0/0	0.063	0/12/11/1	6/1/3/0	<0.001
NAS (0/1/2/3/ 4/5/6/7)	2/2/8/6/ 3/2/0/0	0/7/4/0/ 0/0/0/0	0.010	0/0/0/2/ 14/6/0/2	1/0/0/5/ 4/0/1/0	0.006

VAT – visceral adipose tissue; FBG – fasting blood glucose; LDL – low density lipoprotein; HDL – high density lipoprotein; ALT – alanine transaminase; MV – microvesicular; MDB – Mallory-Denk body; MM megamitochondria; LI – lobular inflammation; PI – portal Inflammation; PK – pigmented Kupffer; PS – perisinusoidal; PP – periportal; NAS – NAFLD activity score

Supplementary Results Table 3. Top 100 DE genes in the DINAH model non-tumour tissues G2-G1 comparison.

symbol	log2FoldChange	pvalue	padj
Rab7b	1.359134316	8.56E-18	1.56E-13
Rgs2	1.326200401	1.17E-16	1.07E-12
Itgax	3.070569217	5.29E-16	3.22E-12
Ephb2	5.037418963	2.23E-15	1.02E-11
Fam83a	2.959473584	3.55E-15	1.30E-11
Ccl5	2.810290262	6.37E-15	1.94E-11
Rgs10	1.259532556	1.08E-14	2.46E-11
Esm1	2.871008568	1.04E-14	2.46E-11
Pparg	4.08289923	1.41E-14	2.86E-11
Arl4c	1.210437925	1.74E-14	3.18E-11
Clec12a	1.382969639	3.41E-14	5.37E-11
Marcks	0.808501292	3.53E-14	5.37E-11
Clec7a	2.06592789	7.76E-14	1.09E-10
Iqgap1	0.95801272	1.06E-13	1.25E-10
Rac2	1.456875313	1.09E-13	1.25E-10
Fabp4	1.014439538	1.03E-13	1.25E-10
Anxa2	1.50396261	1.35E-13	1.45E-10
Epsti1	1.247936037	1.77E-13	1.80E-10
Ncf2	1.091980182	2.08E-13	2.00E-10
Cd300lb	1.141537474	2.32E-13	2.12E-10
Tyrobp	1.152122859	3.40E-13	2.96E-10
Slpi	2.829778489	3.83E-13	3.18E-10
Laptm5	1.241558944	4.21E-13	3.25E-10
Ctss	1.313986275	4.28E-13	3.25E-10
Lgals1	1.750814714	4.44E-13	3.25E-10
S100a11	1.946599023	4.69E-13	3.30E-10
Card11	2.36672697	4.96E-13	3.35E-10
Gltp	0.928456203	9.90E-13	6.46E-10
Timp1	3.260114433	1.19E-12	7.45E-10
Wdfy4	1.155041207	1.22E-12	7.45E-10
Fam105a	1.435609461	1.43E-12	8.42E-10
Rftn1	0.988664999	1.87E-12	1.07E-09
Cd68	0.989247112	2.02E-12	1.09E-09
Vav1	1.133073448	2.03E-12	1.09E-09
Ckb	1.099940398	2.23E-12	1.17E-09
Endod1	0.969749043	2.68E-12	1.36E-09
Thbs1	1.940743741	2.76E-12	1.36E-09
Kbtbd11	1.635367339	3.11E-12	1.49E-09
Nckap1l	1.187748515	3.21E-12	1.51E-09
Arhgap25	1.304362396	4.79E-12	2.19E-09
Cidec	3.185851928	7.45E-12	3.32E-09
Ucp2	1.373021954	7.76E-12	3.36E-09
Msmp	3.396284509	7.90E-12	3.36E-09
Pld4	1.117857684	9.70E-12	4.03E-09
Cotl1	0.814243272	1.06E-11	4.29E-09
NA	-4.079536802	1.65E-11	6.54E-09
Clec4a2	1.672108973	1.72E-11	6.55E-09
5830473C10Rik	-0.688594907	1.72E-11	6.55E-09

Gpr65	1.390705015	1.80E-11	6.70E-09
Pak1	1.338584911	2.24E-11	8.20E-09
Trem2	2.447214461	3.58E-11	1.19E-08
Sash3	1.509624283	3.44E-11	1.19E-08
Rap2b	0.900109359	3.54E-11	1.19E-08
B4galt6	1.151351281	3.45E-11	1.19E-08
Ms4a4b	1.87320898	3.52E-11	1.19E-08
Uhrf1	2.780330841	3.68E-11	1.20E-08
Tnfrsf23	1.382085065	3.86E-11	1.24E-08
Capg	1.36288818	3.98E-11	1.25E-08
Ly6d	3.248552011	4.06E-11	1.26E-08
Gnai1	1.527483917	4.32E-11	1.32E-08
Hr	2.234750158	4.40E-11	1.32E-08
Myl12b	0.495840381	4.56E-11	1.34E-08
Lpxn	1.126618128	5.08E-11	1.47E-08
Dock2	1.317705168	6.39E-11	1.82E-08
Amz1	1.831023718	7.30E-11	2.05E-08
Ly86	1.140937813	9.25E-11	2.52E-08
Hip1	0.687771282	9.17E-11	2.52E-08
Mbp	1.894942589	1.04E-10	2.78E-08
Csf1r	0.974790629	1.18E-10	3.11E-08
Was	1.190245519	1.19E-10	3.11E-08
Cybb	1.316363724	1.36E-10	3.50E-08
Sh3pxd2b	1.216376464	1.43E-10	3.63E-08
Fcer1g	1.039992004	1.70E-10	4.25E-08
Ppm1h	0.98991467	1.81E-10	4.47E-08
Myo1f	1.313352426	2.38E-10	5.79E-08
Ms4a7	2.301540961	2.47E-10	5.85E-08
Acot9	0.928396503	2.46E-10	5.85E-08
Tnfaip8l2	1.176264257	2.67E-10	6.10E-08
Gpsm3	0.784568538	2.66E-10	6.10E-08
Dna2	-0.623830814	2.64E-10	6.10E-08
Cyba	1.06235638	3.02E-10	6.81E-08
H2-M2	3.752112724	3.10E-10	6.92E-08
Cd52	1.801688923	3.19E-10	7.02E-08
Cxcl9	1.874650962	3.61E-10	7.85E-08
Fam129a	1.035352159	3.74E-10	8.03E-08
NA	1.21220214	3.88E-10	8.23E-08
Wisp2	3.191471438	3.98E-10	8.35E-08
Arhgap22	2.827918458	4.11E-10	8.54E-08
Cyth4	1.108570951	4.43E-10	9.10E-08
Ppp4r3a	-0.432994015	4.80E-10	9.74E-08
Myof	1.198870527	4.98E-10	1.00E-07
Trim30d	0.999994332	5.16E-10	1.03E-07
Pdcd1	5.134805636	5.41E-10	1.06E-07
Ccnd1	1.695752427	5.47E-10	1.06E-07
Sh3bgrl3	0.952296721	5.55E-10	1.07E-07
Cd48	1.291535518	6.20E-10	1.18E-07
Slc7a8	0.86707134	6.39E-10	1.20E-07
Rinl	1.15276684	6.55E-10	1.22E-07
Scube1	2.236732896	6.70E-10	1.24E-07
Ifit3b	1.183226877	7.13E-10	1.30E-07

Supplementary References

[1] European Association for the Study of the Liver. Electronic address eee, European Association for the Study of the L. EASL Clinical Practice Guidelines: Management of hepatocellular carcinoma. J Hepatol 2018;69:182-236.

[2] Kleiner DE, Brunt EM, Van Natta M, Behling C, Contos MJ, Cummings OW, et al. Design and validation of a histological scoring system for nonalcoholic fatty liver disease. Hepatology 2005;41:1313-1321.

[3] Gordon H. SHH. A simple method for the silver impregnation of reticulin. American Journal of Pathology 1936;12:545.

[4] Love MI, Huber W, Anders S. Moderated estimation of fold change and dispersion for RNA-seq data with DESeq2. Genome Biol 2014;15:550.

[5] Babicki S, Arndt D, Marcu A, Liang Y, Grant JR, Maciejewski A, et al. Heatmapper: web-enabled heat mapping for all. Nucleic acids research 2016;44:W147-153.

[6] Mootha VK, Lindgren CM, Eriksson KF, Subramanian A, Sihag S, Lehar J, et al. PGC-1alpha-responsive genes involved in oxidative phosphorylation are coordinately downregulated in human diabetes. Nature genetics 2003;34:267-273.

[7] Subramanian A, Tamayo P, Mootha VK, Mukherjee S, Ebert BL, Gillette MA, et al. Gene set enrichment analysis: a knowledge-based approach for interpreting genome-wide expression profiles. Proc Natl Acad Sci U S A 2005;102:15545-15550.

[8] Liberzon A, Subramanian A, Pinchback R, Thorvaldsdottir H, Tamayo P, Mesirov JP. Molecular signatures database (MSigDB) 3.0. Bioinformatics 2011;27:1739-1740.

[9] Erickson BJ, Kirk, S., Lee, Y., Bathe, O., Kearns, M., Gerdes, C., Lemmerman, J. Radiology Data from The Cancer Genome Atlas Liver Hepatocellular Carcinoma [TCGA-LIHC] collection. The Cancer Imaging Archive. 2016.

[10] Schwalbe EC, Williamson D, Lindsey JC, Hamilton D, Ryan SL, Megahed H, et al. DNA methylation profiling of medulloblastoma allows robust subclassification and improved outcome prediction using formalin-fixed biopsies. Acta Neuropathol 2013;125:359-371.

[11] Brunet JP, Tamayo P, Golub TR, Mesirov JP. Metagenes and molecular pattern discovery using matrix factorization. Proceedings of the National Academy of Sciences of the United States of America 2004;101:4164-4169.

[12] Tamayo P, Scanfeld D, Ebert BL, Gillette MA, Roberts CW, Mesirov JP. Metagene projection for cross-platform, cross-species characterization of global transcriptional states. Proceedings of the National Academy of Sciences of the United States of America 2007;104:5959-5964.
